# Mechanistic insights into gut microbiota dysbiosis in osteoarthritis based on the gut-joint axis

**DOI:** 10.3389/fimmu.2026.1892592

**Published:** 2026-09-03

**Authors:** Yingke Liu, Xiaohui Guo, Shu Zhang, Xiaokun Kuang, Jiaxiang Yan, Kewei Tian, Kejie Fan, Wenlong Ma, Ke Chen

**Affiliations:** 1 Rehabilitation Department of Spinal Related Diseases, Luoyang Orthopedic-Traumatological Hospital of Henan Province (Henan Provincial Orthopedic Hospital), Luoyang, China; 2 Rehabilitation Medicine College, Henan University of Chinese Medicine, Zhengzhou, China; 3 Department of Hip Joint Surgical Diagnosis and Treatment Center, Luoyang Orthopedic-Traumatological Hospital of Henan Province (Henan Provincial Orthopedic Hospital), Luoyang, China

**Keywords:** gut microbiota dysbiosis, gut-joint axis, intestinal immune, knee osteoarthritis, osteoarthritis

## Abstract

Osteoarthritis (OA) is a highly prevalent degenerative joint disease characterized by articular cartilage degradation, extracellular matrix disruption, subchondral bone sclerosis, and osteophyte formation, pathological features that collectively impair joint function and significantly reduce patients’ quality of life. Therefore, elucidating the systemic pathogenic mechanisms of OA is essential for enabling early diagnosis and implementing disease modifying interventions. Recent studies demonstrate that the gut microbiota, as a key regulatory factor, actively participates in OA pathogenesis via the bidirectional “gut-joint axis.” Gut microbiota dysbiosis, including depletion of beneficial commensal bacteria, Th17/Treg imbalance, aberrant macrophage polarization, and impaired intestinal barrier integrity, disrupts host microbiota symbiosis and promotes low-grade systemic inflammation, thereby amplifying synovial inflammation, cartilage catabolism, and subchondral bone remodeling. Although accumulating evidence robustly links gut dysbiosis to OA progression, the precise molecular and cellular mechanisms, particularly how specific alterations in gut microbiota composition and metabolites contribute to OA onset and development, remain incompletely defined. This article summarizes current insights into the gut-joint axis in OA and OA-related risk factors, focusing on structural alterations in the gut microbiota and shifts in key metabolites driven by intestinal dysbiosis in OA. Based on this, targeting the gut microbiota is proposed as a promising therapeutic strategy for OA.

## 1 Introduction

Osteoarthritis (OA) is a highly prevalent, progressive degenerative joint disorder characterized pathologically by articular cartilage degradation, chondrocyte dysfunction, subchondral bone sclerosis and osteophyte formation (1). Clinically, it manifests primarily as chronic joint pain, stiffness, and progressive loss of mobility; in advanced stages, it leads to irreversible joint deformity and functional disability—contributing significantly to global morbidity, impaired quality of life, and substantial socioeconomic burden (2). Epidemiological data show that the global prevalence of OA continues to rise. As of 2021, there were approximately 606.5 million OA patients worldwide, and the age-standardized prevalence rate was 6,967.3 cases per 100,000 people (3). It is predicted that the number of patients in 2050 will approach 1 billion (4). Therefore, strengthening OA prevention and early intervention is crucial to reducing the burden of OA. OA pathogenesis is multifactorial, driven by interrelated biological and biomechanical risk factors, including aging, female sex, obesity, metabolic syndrome (MetS), hypertension, occupational or sports-related joint overloading, and prior joint injury or trauma (5, 6). Interestingly, the risk factors for OA (such as aging, obesity, and diet) are related to the intestinal microbiota, which is thought to be involved in the occurrence and development of OA. Given the multifactorial and heterogeneous pathogenesis of OA, current clinical management remains largely symptomatic and palliative, encompassing intra-articular injections (e.g., corticosteroids, hyaluronic acid, and mesenchymal stromal cells), nonsteroidal anti-inflammatory drugs (NSAIDs), and surgical interventions (primarily joint arthroplasty) (7, 8). However, chronic NSAIDs use is associated with serious gastrointestinal, cardiovascular, and renal adverse effects; while joint arthroplasty provides effective pain relief, it carries risks of implant failure, infection, and progression of disease in contralateral joints (3, 6, 8). To date, no disease-modifying osteoarthritis drugs (DMOADs) have been approved; therefore, further elucidation of OA pathogenesis is essential for developing effective, structure-sparing therapies.

OA has long been categorized as a non-inflammatory joint disorder; however, accumulating evidence indicates that chronic low-grade inflammation is a central driver of OA pathogenesis (9). Critically, this inflammatory milieu is mechanistically linked to gut microbiome dysbiosis, a state of compositional and functional imbalance in the intestinal microbial ecosystem (10, 11). The gut microbiota refers to the complex microbial community residing in the human gastrointestinal tract, which maintains a dynamic symbiotic relationship with the host through multiple mechanisms, including regulation of immune responses, production of bioactive metabolites (e.g., short-chain fatty acids (SCFAs), secondary bile acids), and preservation of intestinal barrier integrity (12, 13). In addition, disorder of bacterial community structure can induce chronic inflammation, insulin resistance, and abnormal lipid metabolism and then participate in the pathogenesis of many chronic diseases. In OA patients, metagenomic analyses consistently reveal increased relative abundances of *Actinobacteriota* and *Proteobacteria*, decreased *Bacteroidota* abundance, and an elevated *Firmicutes*/*Bacteroidota* (F/B) ratio (11, 14). Such gut dysbiosis can trigger systemic low-grade inflammation and microbial translocation, accelerating OA progression (15). Moreover, enrichment of specific bacterial taxa—such as *Streptococcus*—was significantly correlated with clinical measures of joint pain and synovial inflammation (10). Importantly, a preclinical study demonstrated that supplementation with specific probiotic *Lactobacillus rhamnosus* suppresses pro-inflammatory cytokine expression, inhibits cartilage catabolism, and attenuates structural damage in OA models (16). Collectively, these findings support a causal, rather than merely associative, link between gut microbiota dysbiosis and OA, suggesting that targeted modulation of the gut microbiome represents a promising therapeutic avenue for OA prevention and disease modification.

Although the pathogenesis of OA remains incompletely elucidated, accumulating evidence supports a pivotal role for the gut–joint axis in skeletal disorders, offering a paradigm-shifting framework for understanding and treating OA (17, 18). The gut–joint axis describes a bidirectional, multi-layered communication network between the intestine and joints, mediated by immune cell trafficking, microbial metabolite circulation (e.g., lipopolysaccharide (LPS), SCFAs), and dynamic alterations in intestinal barrier permeability (18). Gut microbiota dysbiosis has been shown to initiate or exacerbate joint inflammation, through mechanisms including Th17/Treg imbalance and macrophage activation, whereas microbial eubiosis is essential for maintaining systemic immune homeostasis and metabolic equilibrium (12, 19). Moreover, gut-derived microbial metabolites, particularly LPS, can translocate across a compromised epithelial barrier and directly activate TLR4/NF-κB signaling in synovial and chondral tissues, disrupting local redox and matrix homeostasis and ultimately driving structural joint damage (20). Consequently, deciphering the core molecular mediators and causal pathways of the gut–joint axis in OA represents a research objective with direct therapeutic implications (21, 22). This review synthesizes current evidence on the gut–joint axis in OA pathogenesis and clarifies the associations between OA risk factors and intestinal microbiota dysbiosis. Subsequently, it highlights the mechanistic contributions of gut microbiota dysbiosis (especially changes in the composition of the gut microbiota) and its functional metabolites and proposes targeted modulation of the gut microbiome as a rational strategy for disease modification. In short, grounded in the gut–joint axis framework, therapeutic interventions must address both intestinal microbiota dysbiosis and modifiable upstream risk factors, such as obesity, dietary imbalance, and aging, to improve long-term outcomes and advance personalized OA management.

## 2 Current perspectives on the gut–joint axis

Clinical and preclinical evidence from OA indicates that intestinal dysbiosis often precedes disease onset and modulates disease progression (11, 23, 24). Consequently, elucidating the immunological crosstalk between the gut microbiota and joints—the so-called “gut–joint axis”—has emerged as a key focus in musculoskeletal research (18). The intestinal microbiome is a dynamic, host–microbe ecosystem that actively regulates diverse physiological and pathological processes, including immune homeostasis, metabolism, neuroendocrine signaling, and systemic inflammation (25, 26). It engages in bidirectional communication with multiple organs via dedicated microbial–host axis, collectively termed “gut–organ axis”—such as the gut–brain, gut–kidney, gut–liver, gut–bone, and gut–joint axis (27–29). By fecal microbiota transplantation (FMT) from individuals with metabolic dysfunction into germ-free mice subjected to meniscal and ligamentous injury (MLI) surgery, recipient mice exhibited significantly increased relative abundances of *Fusobacterium* and *Faecalibaterium* and significantly decreased relative abundance of *Ruminococcaceae* and increased intestinal permeability, both of which were positively correlated with OA severity (30). This further suggests a clear association between gut microbiota dysbiosis and OA. In the context of OA, cross-organ regulation involves the transmission of microbe-derived inflammatory mediators (e.g., LPS, SCFAs) and immune cell signals from the gut to the joint, thereby triggering local synovial inflammation, cartilage degradation, and subchondral bone remodeling (31). Accumulating preclinical and clinical data robustly link gut microbiota dysbiosis and altered microbial metabolite profiles, including reduced SCFAs and elevated LPS, to OA pathogenesis, substantiating the biological plausibility and translational relevance of the gut–joint axis.

**Figure 1:**
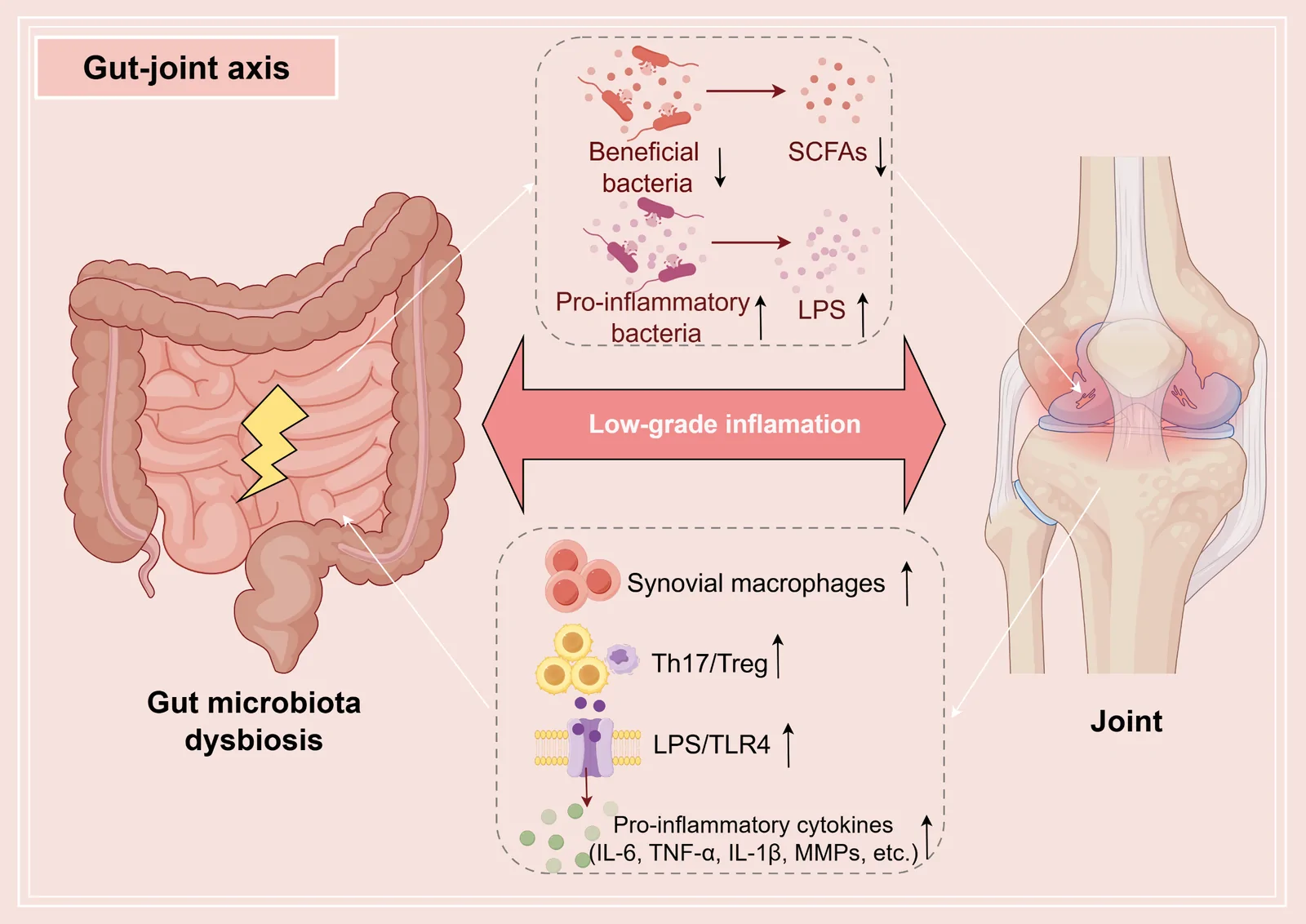
Mechanism of the gut-joint axis in OA. Patients with osteoarthritis (OA) often develop intestinal flora disorders before the onset of the disease, promoting disease progression. Dysfunction of the intestinal flora leads to a decrease in beneficial bacteria, especially short-chain fatty acids (SCFAs)-producing bacteria. Besides, increased intestinal permeability and an increase in pro-inflammatory bacteria lead to enhanced release of lipopolysaccharide (LPS), which activates the LPS/TLR4/NF-κB pathway and promotes the release of pro-inflammatory cytokines (e.g., TNF-α, IL-1β, and IL-6). At the same time, OA is accompanied by expansion of Th17 cells and synovial macrophages, as well as a deficiency in regulatory T cells (Tregs), resulting in an imbalance in the Th17/Treg ratio, which in turn triggers local and systemic inflammation. Taken together, these events maintain the low-grade systemic inflammatory state in patients with OA.

A joint is a dynamic structural unit between bones that mediates mechanical load transmission, motion articulation, and proprioceptive sensing. Its core anatomical components comprise the articular surface, covered by hyaline cartilage, the fibrous-synovial double-layered joint capsule, and the sealed joint cavity containing synovial fluid. Traditionally, the joint cavity has been regarded as a sterile microenvironment; however, accumulating evidence challenges this notion: in OA, subchondral bone–derived biochemical factors can translocate across the pathologically remodeled osteochondral interface to the overlying articular cartilage (32–34). Histopathological studies consistently demonstrate microfractures at the osteochondral junction in OA joints. These structural disruptions promote pathological angiogenesis, thereby establishing a conduit for blood-derived inflammatory mediators (e.g., tumor necrosis factor-α (TNF–α), interleukin (IL)-1β, and IL–6), microbial metabolites, and signaling molecules to infiltrate the joint space (35–37). This process is mechanistically linked to gut dysbiosis—characterized by reduced microbial diversity, depletion of SCFAs–producing bacteria, and increased intestinal permeability. Dysbiosis activates the LPS/Toll-like receptor 4 (TLR4)/nuclear factor kappa-light-chain-enhancer of activated B cells (NF–κB) pathway, elevating systemic levels of pro–inflammatory cytokines, including sCD14, TNF–α, IL–1β, IL–6, and IL–8, and compromising intestinal epithelial tight junction integrity, thereby facilitating translocation of pathogen–associated molecular patterns (PAMPs) (17, 38, 39). Concurrently, OA is associated with Th17 cell and synovial macrophage expansion alongside Treg cell deficiency, resulting in a skewed Th17/Treg ratio that drives local and systemic inflammation and accelerates cartilage degradation (40–42) (**Figure 1**). Collectively, these events sustain low–grade systemic inflammation, which exacerbates OA pathogenesis via upregulation of pro–inflammatory cytokines and matrix metalloproteinases (MMPs), particularly MMP–1 and MMP–13. Consequently, gut microbiota dysregulation impairs intestinal endocrine signaling, mucosal immunity, and barrier function; alters host hormone and cytokine secretion; and promotes systemic dissemination of microbes, microbial components, and inflammatory metabolites. Such disturbances disrupt both systemic and joint–local homeostasis, contribute to metabolic dysfunction and obesity, and ultimately fuel OA initiation and progression.

The concept of the gut–joint axis aims to systematically elucidate the pathophysiological links among gut microbiota dysbiosis, the systemic immune–inflammatory response it drives, and OA pathogenesis. As mechanistic research advances, key microbe–host interaction nodes within this axis are emerging as promising therapeutic targets, offering disease-modifying treatment strategies for OA patients who rely long-term on pharmacological symptom control.

## 3 Risk factors for OA

**Figure 2:**
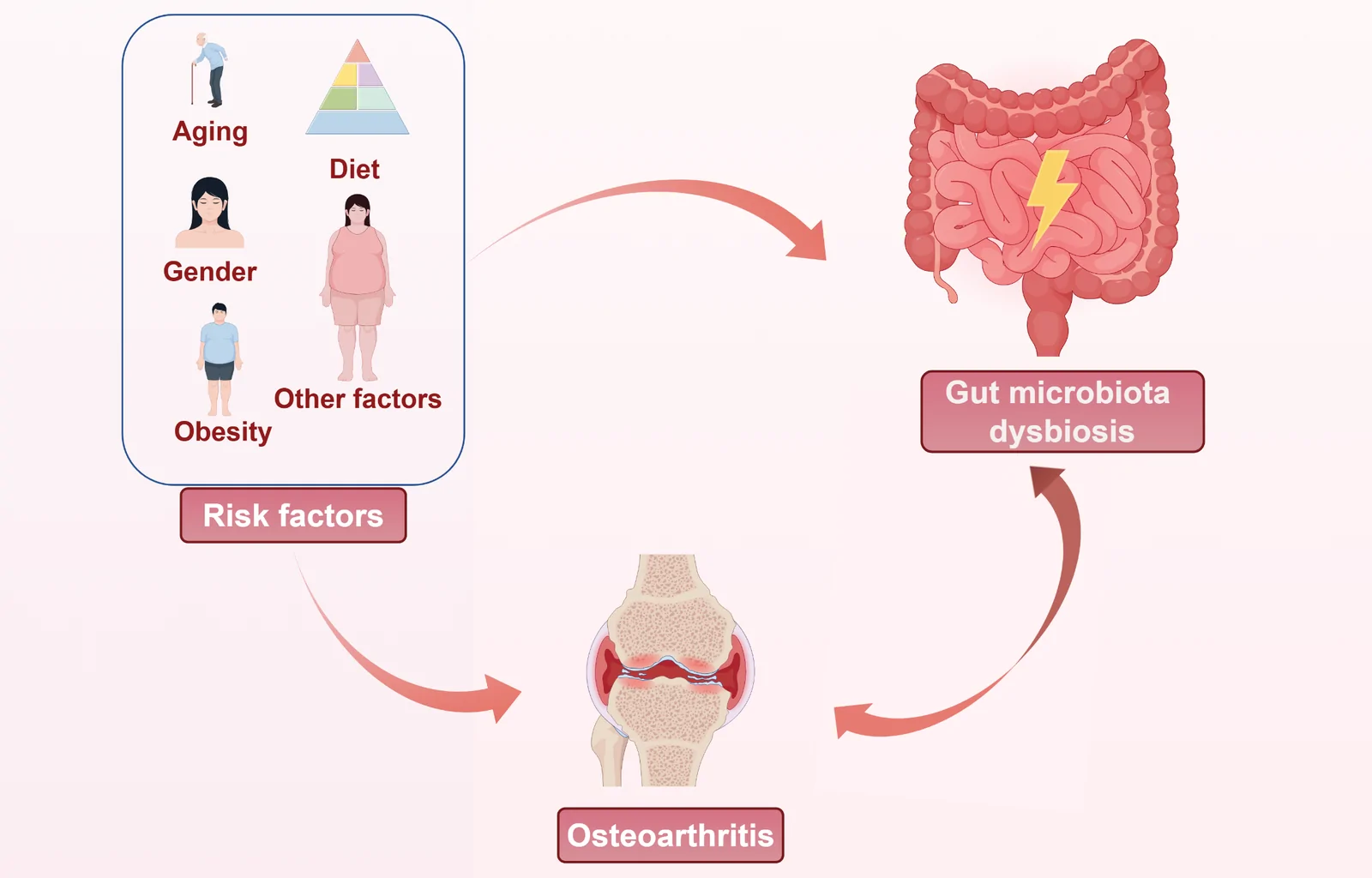
Risk factors for osteoarthritis (OA). Risk factors for OA indirectly promote OA onset and progression by inducing intestinal dysbiosis, or directly contribute to OA onset and progression.

OA is not merely a simple joint degenerative disease in traditional understanding but rather a chronic inflammatory joint disorder mediated significantly by gut microbiota dysbiosis and driven by multiple modifiable and non-modifiable risk factors (43). Multiple modifiable (e.g., obesity and joint trauma) and non-modifiable (e.g., aging and genetic susceptibility) risk factors do not act in isolation; instead, they disrupt host–microbiota homeostasis, particularly inducing gut microbiota dysbiosis, thereby activating key inflammatory pathways such as TLR4/NF-κB, the complement system, and the NOD-like receptor protein 3 (NLRP3) inflammasome, ultimately driving chronic low-grade inflammation, which collectively promotes OA onset and progression. In addition, other diseases may also directly affect joints, thereby promoting the development or progression of OA. Therefore, clarifying the risk factors for OA can inform evidence-based strategies for early prevention and personalized treatment management (**Figure 2**).

### 3.1 Aging

The prevalence of OA increases with age, indicating that aging is a major risk factor. In two separate systematic reviews and meta-analyses focusing on knee osteoarthritis (KOA) and hip osteoarthritis (HOA), respectively, researchers consistently identified aging as a well-established risk factor—a finding corroborated by multiple independent studies (44–47). To elucidate the role of aging in OA pathogenesis, multiple mechanistic pathways have been proposed (48). First, chondrocytes—the sole resident cell type in articular cartilage—undergo senescence, a process now established as a central driver of OA progression. Hallmark features include irreversible cell cycle arrest, enhanced resistance to apoptosis, and sustained secretion of the senescence-associated secretory phenotypes (SASPs), encompassing IL-1β, TNF-α, IL-6, and MMPs (49, 50). Second, aging-associated mitochondrial dysfunction triggers oxidative stress, leading to increased generation of reactive oxygen species (ROS) and elevated production of pro-inflammatory cytokines, thereby exacerbating local joint inflammation (51, 52). Third, mitochondrial damage activates the NLRP3 inflammasome, resulting in robust release of IL-1β and TNF-α—key mediators that amplify chondrocyte-intrinsic inflammation and accelerate apoptotic cell death (53, 54). Importantly, aging can impair intestinal barrier function, characterized by increased intestinal permeability and secondary gut microbiota dysbiosis. Specifically, gut-derived LPS translocates across the compromised intestinal barrier and binds specifically to TLR4 on macrophages, driving their polarization toward the classically activated M1 phenotype, thereby activating inflammatory signaling pathways such as NF-κB and amplifying both local and systemic inflammation (55, 56). Therefore, the aging process disrupts intestinal microbial homeostasis, compromises gut barrier integrity, and dysregulates both innate and adaptive immune responses, collectively fostering a pro-inflammatory milieu that accelerates the onset and progression of OA.

### 3.2 Gender

OA exhibits a higher prevalence in women than in men, particularly among older adults (57). Moreover, female patients with OA frequently report more severe joint pain and greater functional impairment compared with their male counterparts (58). Women’s higher risk of OA may stem from sex-specific differences in joint anatomy, alignment, muscle strength, hormonal influences, and obesity. At equivalent radiographic severity, women report greater pain intensity than men, a disparity potentially attributable to biologically distinct pain-processing mechanisms, differential engagement of central pain-modulatory pathways, and variations in pain sensitivity and coping strategies (59, 60). Besides, estrogen promotes chondrocyte synthesis of proteoglycans and synergistically suppresses the expression of key pro-inflammatory and catabolic mediators, including cyclooxygenase-2 (COX-2), ROS, inducible nitric oxide synthase (iNOS), and NF-κB, thereby significantly attenuating cartilage degeneration and inhibiting pathological bone remodeling and osteophyte formation (61, 62). This may be related to estrogen enhancing women’s susceptibility to inflammatory stimuli and their associated immune responses (63). After menopause, estrogen levels in women significantly decline, and *Prevotella* may have a protective effect against bone loss in postmenopausal women (64). Interesting, sex is one of the major factors influencing intestinal microbiota composition (65). Sex hormone deficiency may increase intestinal permeability (66), and sex-based differences in gut microbial composition are likely mediated by sex hormones. However, no study has yet established a causal link between these gender-associated microbial differences and the higher prevalence of osteoarthritis in women. Future research should directly compare the gut microbiota profiles of female and male OA patients to elucidate the underlying mechanisms contributing to the elevated incidence in women. Therefore, clarifying the gender-specific gut characteristics of OA can provide a key basis for individualized, gender-appropriate diagnosis and treatment strategies.

### 3.3 Obesity

Overweight and obesity have been widely recognized as important controllable risk factors for OA, especially KOA (67, 68). Epidemiological studies have shown that for every 5 kg increase in body weight, the probability of developing KOA increases by 36% (69). In a systematic review and meta-analysis of KOA, researchers found that a high body mass index (BMI) was a significant risk factor (46). Weight loss interventions significantly improve clinical symptoms in patients with obesity-related OA (70), an effect that may be mechanistically linked to reductions in total adipose tissue mass (71). Adipose tissue secretes pro-inflammatory cytokines through TLR4/NF-κB pathways—including TNF-α, IL-6, and IL-1β—thereby sustaining systemic low-grade inflammation. Following weight loss, this inflammatory burden diminishes, leading to attenuated inflammatory damage of articular cartilage (72). Studies have demonstrated that high-fat diet intervention in mice elevates the F/B ratio, inducing gut microbiota dysbiosis and compromising intestinal mucosal barrier integrity. This increased intestinal permeability permits bacterial endotoxins such as LPS to translocate, thereby activating synovial macrophages and promoting their polarization toward a pro-inflammatory phenotype, which exacerbates local joint inflammation and ultimately accelerates OA progression (73–75). At the same time, obesity-related OA is associated with reduced abundance of Bifidobacterium and increased abundance of pro-inflammatory bacteria (76). In addition, obesity increases mechanical loading on the joints, subjecting cartilage tissue to sustained abnormal pressure; this, in turn, accelerates degradation of the cartilage extracellular matrix and triggers the SASPs (77). Therefore, weight management and dietary optimization are effective strategies for reducing the risk of OA.

### 3.4 Diet

Clinical epidemiological evidence confirms that dietary patterns represent a key risk factor influencing both the incidence and progression of OA (78, 79). For example, studies have demonstrated that a high-carbohydrate, high-fat, or high-protein dietary pattern, particularly one rich in refined sugars and saturated fatty acids, synergistically activates systemic inflammatory pathways and directly exacerbates both metabolic dysregulation and structural degeneration of articular cartilage (80, 81). Interestingly, diets high in sugar and fat or low in dietary fiber impair intestinal barrier function in mice and promote microbial translocation, a process potentially linked to OA progression (82, 83). In contrast, the Mediterranean dietary pattern and high-fiber dietary interventions have been consistently demonstrated to suppress systemic inflammation, preserve cartilage homeostasis, and significantly alleviate clinical symptoms in patients with OA (84). Interestingly, individuals with higher dietary index for gut microbiota (DI-GM) scores, a metric designed to assess how dietary patterns influence gut microbiome health, exhibited a significantly lower risk of developing OA (85). Therefore, diet should be included in the daily comprehensive treatment strategy of OA.

### 3.5 Other factors

Interestingly, other diseases can also be predisposing factors for OA. Based on data from the China Health and Retirement Longitudinal Study (CHARLS), a cohort analysis of 6,212 Chinese older adults revealed that individuals with sarcopenia had a nearly twofold increased risk of developing KOA compared with those without sarcopenia (86). Sarcopenia is characterized by a higher abundance of *Proteobacteria* at the phylum level, a lower abundance of *Firmicutes*, an increased abundance of *Escherichia-Shigella* at the genus level, and reduced abundances of *Prevotella* and *Blautia* (87). Therefore, alterations in the gut microbiota may contribute to the increased susceptibility of sarcopenia to KOA. Additionally, existing evidence shows that MetS and its core components (such as hypertension, central obesity, elevated blood glucose, and dyslipidemia) are significantly associated with an increased risk of OA and its symptomatic progression, suggesting that OA is not merely a degenerative joint disease but rather a disorder with a well-established metabolic-inflammatory basis (88). More and more studies indicate that MetS is associated with distinct gut microbiota alterations, characterized primarily by the overgrowth of harmful bacteria and depletion of beneficial bacteria, leading to intestinal barrier dysfunction (89, 90). This may represent one of the key mechanisms by which MetS promotes OA progression.

## 4 Gut microbiota dysbiosis in OA

**Figure 3:**
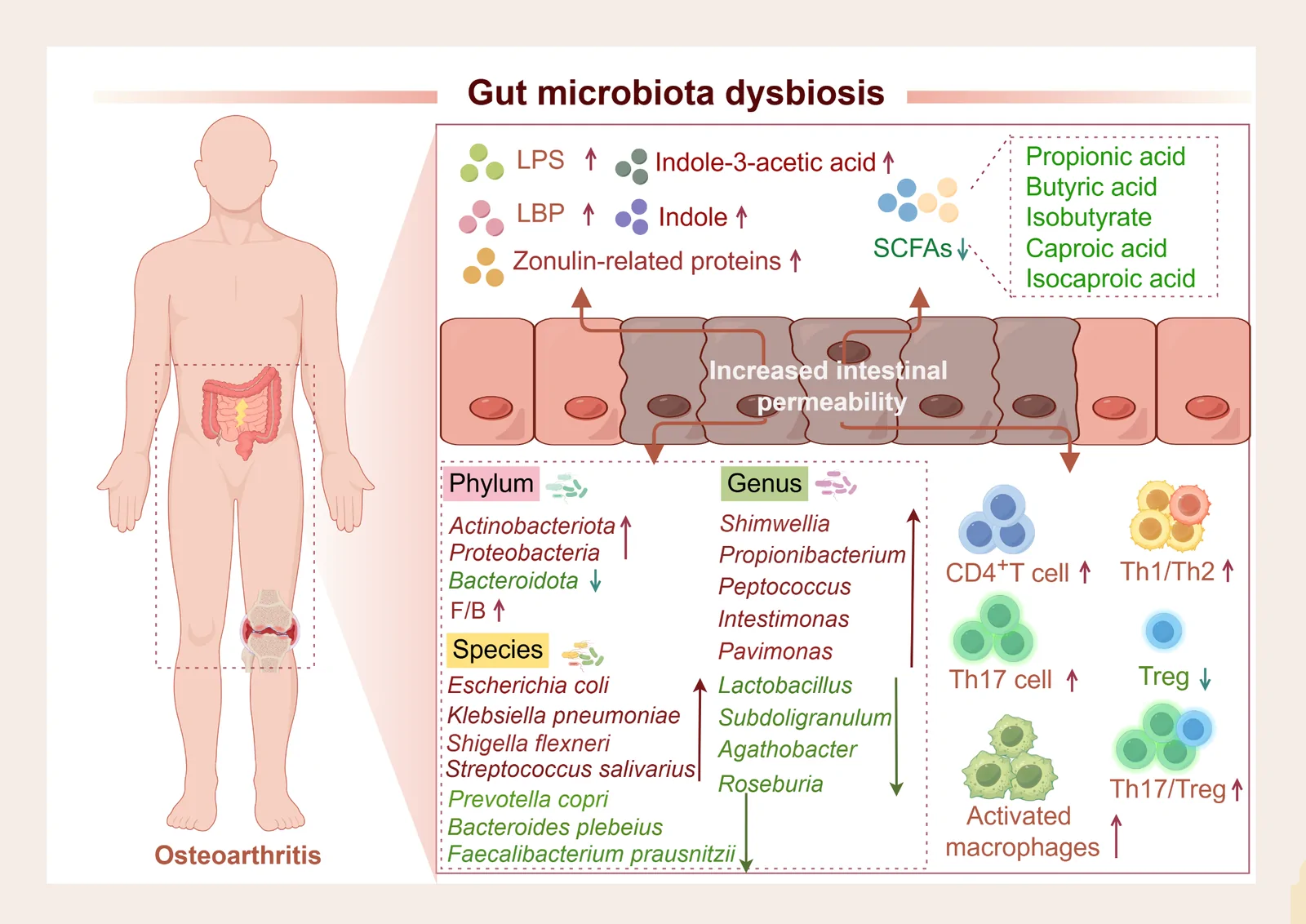
Gut microbiota dysbiosis in patients with osteoarthritis (OA). OA is associated with gut microbiota dysbiosis (at the phylum, genus, and species levels) and increased intestinal permeability. Specifically, there is a decrease in beneficial bacteria and an increase in pro-inflammatory bacteria, along with reduced levels of short-chain fatty acids (SCFAs), including propionic acid, butyric acid, isobutyric acid, caproic acid, and isocaproic acid. Concurrently, increased intestinal permeability leads to elevated levels of lipopolysaccharide (LPS), LPS-binding protein (LBP), and Zonulin-related proteins. Moreover, intestinal flora–mediated tryptophan metabolites—such as indole and indole-3-acetic acid—are also increased. Furthermore, OA is accompanied by expansion of CD4^+^ T cells and Th17 cells, activation of macrophages, and a deficiency in regulatory T cells (Tregs), resulting in imbalances in the Th17/Treg and Th1/Th2 ratios, which in turn trigger local and systemic inflammation.

The gut microbiota denotes the highly diverse and dynamic microbial community inhabiting the human gastrointestinal tract. It engages in a bidirectional, symbiotic relationship with the host—mediated through multiple interdependent mechanisms, including immunomodulation, biosynthesis of bioactive metabolites, and maintenance of intestinal epithelial barrier integrity. In patients with OA, gut microbiota dysbiosis is consistently observed, characterized by compositional alterations across taxonomic levels (phylum, genus, and species) and functional impairment, notably increased intestinal permeability resulting from compromised mucosal barrier function (**Figure 3**). Emerging evidence further supports a gut–joint axis, whereby microbial dysregulation contributes to OA pathogenesis and progression. Therefore, clarifying the specific changes in the gut microbiota of patients with OA is crucial for targeting the intestinal flora for the treatment of OA.

### 4.1 The intestinal microbiota in OA

Substantial evidence indicates that gut microbiota dysbiosis in OA patients is closely associated with key OA-related risk factors, including aging, unhealthy dietary patterns, and obesity, and contributes to systemic low-grade inflammation, thereby establishing a mechanistic link between intestinal dysbiosis and OA pathogenesis. Metagenomic profiling of OA patients consistently shows a microbial signature characterized by enrichment of *Actinobacteriota* and *Proteobacteria*, depletion of *Bacteroidota*, and a significantly higher F/B ratio (11, 91). In the gut microbiota of patients with OA, the relative abundances of class *Bacteroidia* and order *Bacteroidales* were significantly increased, whereas the species *Prevotella copri* was significantly decreased (92). Notably, *Prevotella* abundance demonstrated discriminative capacity between OA patients and healthy controls. The potential of *Prevotella copri* to inhibit the progression of OA was demonstrated in another study (93). Therefore, *Prevotella copri* may exert protective effects in OA and represents a promising candidate diagnostic biomarker for OA. At the genus level, the abundances of *Shimwellia*, *Propionibacterium*, *Peptococcus*, *Intestimonas*, and *Pavimonas* were significantly increased in patients with KOA (94). A separate investigation reported marked reductions in the relative abundances of beneficial SCFAs–producing genera in individuals with KOA, including *Agathobacter*, *Subdoligranulum*, *Roseburia*, and *Lactobacillus*, while several taxa, namely *Prevotella* sp. *7*, *Clostridium*, *Flavonifractor*, and *Klebsiella*, showed significant enrichment at the genus level (95). In northwest China, the relative abundance of *Citrobacter*, *Flavonifractor*, *Epsilonbacteraeota*, *Raoultella*, and *Firmicutes* in the intestines of patients with KOA is increased (96). Metagenomic sequencing revealed a marked depletion of several *Bacteroides* species, including *B. plebeius*, *B. stercoris*, *B. vulgatus*, and *B. uniformis*, while opportunistic or inflammation-associated taxa such as *Escherichia coli*, *Klebsiella pneumoniae*, *Shigella flexneri*, and *Streptococcus salivarius* were significantly enriched in OA patients (95, 97). In addition, gut microbiota β diversity—an ecological metric reflecting inter-sample differences in community composition—was significantly altered in patients with symptomatic hand OA; further analysis revealed that this structural shift was closely associated with imbalances in the abundances of functionally relevant bacterial genera: specifically, the relative abundances of *Bilophila* and *Desulfovibrio* were significantly elevated, whereas that of the key butyrate-producing genus *Roseburia* was significantly reduced (98).

In the rat model of PTOA, the relative abundance of the phylum *Fusobacteria* was significantly increased; at the genus level, the relative abundances of *Lactobacillus*, *Turicibacter*, *Adlercreutzia*, and *Cetobacterium* were significantly increased (99). In the anterior cruciate ligament transection (ACLT)-induced murine OA model, the relative abundances of *Proteobacteria* and *Ruminococcus* (a genus within the *Firmicutes* phylum) were significantly elevated (100). In the ACLT-induced OA rat model, the abundances of *Ruminococcus*, *Prevotella*, *Shigella*, *Helicobacter*, *Desulfovibrio*, and *Streptococcus* were significantly also elevated at the genus level (101). However, in a rhesus monkey model of spontaneous OA, the relative abundances of *Prevotella* and *Ruminococcus* were decreased, whereas *Lactobacillus* abundance was increased (102). However, in a rat model of metabolic KOA induced by a high-fat, high-sugar diet (HFS), decreased cecal abundances of *Lactobacillus* were observed (103). The differences in results may be due to the inherent biological characteristics of different animal models. Firstly, metabolism-associated OA models are predominantly established via high-fat diet feeding, commonly exhibiting obesity, insulin resistance, and low-grade inflammation; this metabolic dysregulation directly suppresses *Lactobacillus* species. In contrast, PTOA models are typically induced by mechanical or surgical interventions, whereas spontaneous OA models, such as the rhesus monkey model, are linked to age-related gut microbiota remodeling. Consequently, substantial inter-model differences exist in host species, induction modalities, and baseline metabolic status. Moreover, sequencing methodologies vary across studies: although most employ 16S rRNA gene sequencing, discrepancies in reference database versions, OTU or ASV clustering algorithms, and taxonomic annotation pipelines lead to markedly inconsistent species-level resolution within the genus *Lactobacillus*. Furthermore, nearly all studies report only relative abundance; thus, when abundances of other dominant taxa decline, *Lactobacillus* relative abundance may increase. Critically, the profound etiological heterogeneity of OA dictates that patterns of gut dysbiosis are inherently subtype-specific. Therefore, developing individualized therapeutic strategies for OA patients requires integration of etiological classification with host metabolic phenotype, phylogenetic background, methodological constraints of microbiota profiling, and functional microbial traits. Future studies must combine species-level precise identification, absolute quantification (e.g., via qPCR or metagenomic sequencing), and functional validation to enable robust, multi-dimensional assessment. Meanwhile, it is necessary to systematically compare the composition and functional characteristics of the intestinal microbiota in various OA animal models to identify microbial markers with greater disease specificity and mechanistic relevance.

Dysbiosis-associated expansions of *Fusobacterium* and *Faecalibacterium*, alongside a depletion of *Ruminococcaceae* family members, were significantly correlated with increased radiographic severity and synovial inflammation in OA, suggesting a potential mechanistic contribution of these taxa to disease progression (30). Similarly, an increased relative abundance of *Faecalibacterium* has been reported in patients with adolescent idiopathic scoliosis (104). The relative abundance of *Faecalibacterium* is positively correlated with the histopathological severity of OA and with systemic levels of inflammatory biomarkers. However, at the species level, the abundances of *Faecalibacterium prausnitzii* were significantly reduced in the intestines of patients with OA; this bacterium is a representative beneficial bacteria exhibiting anti-inflammatory and immunoregulatory properties (97). Similarly, in a rat model of metabolic KOA induced by HFS, decreased cecal abundances of *Faecalibacterium prausnitzii* were observed, whereas *Enterobacteriaceae* abundance was increased (103). The apparent contradiction may arise because the *Faecalibacterium* genus comprises multiple species, and its genus-level relative abundance represents the sum of abundances across all constituent species; in contrast, *Faecalibacterium prausnitzii*, though the most representative butyrate-producing species, is functionally distinct. When *Faecalibacterium prausnitzii* abundance declines due to inflammation, oxidative stress, or intestinal microenvironment disruption, other *Faecalibacterium* species may undergo compensatory proliferation and colonize their ecological niche, thereby elevating total genus-level abundance. However, this increase does not reflect enhanced butyrate synthesis capacity or anti-inflammatory functionality. Therefore, in OA gut microbiota research, genus-level abundance alone is insufficient to infer microbial functional propensity (i.e., beneficial versus detrimental effects); instead, integrated multi-omics assessment, including metagenomic sequencing, functional gene profiling, and quantification of key metabolites (e.g., fecal butyrate concentration), is warranted.

### 4.2 Intestinal metabolites in OA

#### 4.2.1 LPS

In view of the intestinal dysbiosis associated with OA, gut-derived metabolites also play an important role in the onset and progression of OA. Impaired intestinal mucosal barrier function enables LPS (products of intestinal bacteria) to breach the intestinal epithelial barrier and enter the systemic circulation via the portal vein system—a process termed “microbial translocation.” This event serves not only as a critical trigger of host innate immunity but also as a key driver of systemic low-grade inflammation, thereby exerting pathological effects on multiple distal organs, including synovial joints (56). As a canonical ligand for the innate immune system, LPS primarily activates downstream inflammatory signaling pathways by binding to the TLR4. Clinical studies further demonstrate that serum LPS concentrations in OA patients correlate significantly with both radiographic severity and clinical symptom burden; synovial fluid LPS levels are strongly associated with joint space narrowing and pain/function impairment (105). Beyond LPS, lipopolysaccharide-binding protein (LBP)—a prototypical acute-phase protein—is established as a key biomarker of OA progression: elevated serum LBP levels show significant associations with both structural deterioration and symptomatic severity in KOA (20). LBP expression is dually regulated by proinflammatory cytokines such as IL-6 and by LPS itself. In addition, serum and synovial fluid levels of LPS and LBP in patients with KOA were significantly positively correlated with the abundance of activated macrophages in the synovium (74). LPS levels were also significantly associated with knee osteophyte formation. This suggests that serum and synovial fluid levels of LPS and LBP are closely associated with OA disease progression and related clinical symptoms; therefore, therapeutic strategies targeting LPS translocation or LBP activity hold promise for OA treatment. Moreover, in a DIGICOD cohort study of 410 patients with hand OA, serum levels of LBP and Zonulin-related proteins were significantly elevated, indicating increased intestinal permeability in patients with hand OA (106). A recent study reported that LBP was significantly positively correlated with the severity of synovitis in patients with obesity-related KOA, and its serum levels could be used to predict severe synovitis (107). Interestingly, LBP and CD14, as key co-receptors of the TLRs signaling pathway, mediate and amplify the cartilage damage process of post-traumatic OA in a low-grade inflammatory environment (108). The absence of both can significantly alleviate this pathological process. The above research findings collectively suggest that targeting the LPS/TLRs relative signaling pathway holds therapeutic potential for OA.

#### 4.2.2 SCFAs

SCFAs—notably acetate, propionate, and butyrate—are major bioactive compounds synthesized by the gut microbiota and play a key role in maintaining host intestinal health (109). The progression of OA is often accompanied by gut microbiota dysbiosis, primarily driven by increased intestinal permeability and decreased abundance of SCFAs-producing bacteria (18). Based on the gut–joint axis mechanism, reduced SCFAs production promotes intestinal inflammation and exacerbates intestinal hyperpermeability, thereby contributing to joint damage (110). In an animal study evaluating the role of intestinal microbiota in obese and non-obese osteoarthritis patients, it was found that intestinal microbiota from non-obese OA patients exacerbated disease progression in ACLT-induced OA rats by increasing acetate levels and decreasing propionate levels (111). In another study, it was also confirmed that acetic acid intervention can aggravate gouty arthritis by lowering the pH of the joint microenvironment and destroying the integrity of the joint structure (112). This suggests that acetate may function as a pro-inflammatory metabolite that actively contributes to the pathogenesis and progression of OA. However, propionate supplementation significantly inhibited the progression of OA (113). Propionic acid exerts epigenetic regulatory effects by targeting histone deacetylase (HDAC) in T cells, thereby promoting the functional activation of Tregs and upregulating the expression of key anti-inflammatory mediators such as IL-10 (114, 115). In the cartilage tissue microenvironment, butyrate, acting primarily through the G-protein-coupled receptors 43 (GPR43), negatively regulates the synthesis and release of pro-inflammatory cytokines by chondrocytes (116). Notably, GPR41, a high-affinity SCFAs receptor, is widely expressed in peripheral sensory ganglia and sympathetic neurons; experimental evidence indicates that it is specifically activated by propionate, leading to modulation of neuronal excitability and synaptic neurotransmitter dynamics (117). Therefore, propionate may coordinately engage the GPR41/GPR43 signaling axis in both chondrocytes and sensory neurons to concurrently exert anti-inflammatory and analgesic effects. However, in a monoiodoacetate (MIA)-induced rat model of OA, acetate and propionate levels in osteoarthritis rats were significantly reduced (118). Similarly, in the MIA-induced rat model of OA, levels of SCFAs, including acetic acid, propionic acid, butyric acid, isobutyric acid, isovaleric acid, caproic acid, isocaproic acid and valeric acid, were significantly decreased (119). At the same time, in a medial meniscus destabilization (DMM) surgery-induced OA mouse model, levels of acetic acid, propionic acid, butyric acid, and isobutyric acid were significantly decreased, whereas levels of isovaleric acid and valeric acid were significantly increased (120). Differences in acetate levels may stem from variations in animal model construction; therefore, future studies should perform refined classification of the gut microbiota and SCFAs in OA subtypes defined by distinct etiologies. When the structure of the intestinal microbiota is disrupted under OA pathology (e.g., decreased abundance of SCFAs-producing bacterial taxa), overall SCFAs levels are significantly reduced, leading to downregulation of tight junction protein expression, increased intestinal permeability, and microbial translocation, thereby exacerbating joint inflammation. Therefore, reduced SCFAs levels are not only a biomarker of intestinal microecological dysbiosis in OA but also a key mediator driving abnormal activation of the gut–joint axis and thereby exacerbating local joint inflammation and structural damage.

#### 4.2.3 Other metabolites

Secondary bile acids are products of primary bile acids metabolized and transformed by gut bacteria. Studies have shown that secondary bile acid levels, particularly HDCA and DCA, are significantly reduced in a rat model of KOA (121). In addition, HDCA supplementation inhibits M1 macrophage polarization while promoting M2 macrophage polarization, thereby delaying KOA progression. In the PFOS-induced OA model, intestinal microbiota dysbiosis was observed, leading to further reduction in secondary bile acid production (122). This bile acid deficiency may impair activation of the farnesoid X receptor (FXR) and its downstream glucagon-like peptide-1 receptor (GLP-1R) in chondrocytes, thereby promoting OA pathogenesis. Therefore, reduced secondary bile acid levels result from gut microbiota dysbiosis in OA patients. Targeted supplementation of deficient secondary bile acids represents a promising therapeutic strategy for OA intervention. Besides, metabolomic analysis of fecal samples from patients with obesity-related OA revealed decreased levels of propionate, suberate, and hippuric acid, and increased levels of indole, indole-3-acetate, and phenylacetate (123). Interestingly, a related study has found that serum levels of two key metabolites, pyrogallol and 3-hydroxybutyric acid, were significantly lower in patients with OA than in healthy controls (97).

### 4.3 Immune cell in OA

#### 4.3.1 T cells

As a key cell type in the adaptive immune response, T cells can secrete a large number of pro-inflammatory cytokines after being specifically activated in the pathological microenvironment (40, 124). Studies have shown that the activation state of T lymphocytes is closely related to the onset and progression of OA (125, 126). Among these, pro-inflammatory cytokines such as IL-1β, TNF-α, and IL-6 participate in the pathogenesis of OA, promote chronic low-grade inflammation, and exacerbate OA progression (71). Existing studies have found that T lymphocytes and macrophages (including synovial tissue-resident macrophages, STRM) constitute the core immune infiltrating components in the synovial tissue and joint synovial fluid of patients with OA (127, 128). Moreover, a large number of CD4^+^ T cells can be detected in the synovium of patients with OA, and the number of Th1 cells is significantly greater than that of Th2 cells (129). Similarly, researchers detected only low levels of IL-4 and IL-10 in the peripheral blood and synovial fluid of patients with OA (130). Importantly, during the early stages of the disease, activated CD4^+^ T cells release substantial amounts of TNF-α and IL-6, thereby driving local synovial inflammation (131). The researchers isolated CD4^+^ T cells from the peripheral blood of patients with KOA, specifically induced their differentiation into Th1, Th2, Th17, and Treg subsets, and then co-cultured these T cell subsets with chondrocytes (132). Results showed that compared to monoculture, the levels of pro-inflammatory factors—MMP-1, MMP-9, MMP-13, and IL-6—secreted by chondrocytes were significantly elevated when co-cultured with each CD4^+^ T cell subset, with the most pronounced increase observed in the Th17 co-culture group. IFN-γ levels increased significantly only in the Th1 co-culture system, while TNF-α secretion was notably reduced only under Treg co-culture conditions compared to monoculture. In patients with OA, both the number of Th17 cells and serum IL-17 levels were significantly elevated (133). IL-17 exacerbates joint inflammation by inducing inflammatory cytokines—including TNF-α, IL-1β, and IL-6—as well as MMPs, thereby contributing to joint structural damage (134). Moreover, studies have reported that IL-17 concentrations in the serum and knee synovial fluid of patients with KOA are positively correlated with disease severity (135, 136). Therefore, the frequency of Th17 cells and their secreted IL-17 levels are considered potential biomarkers of OA disease severity. In summary, Th2 and Treg cell subsets exert anti-inflammatory effects by secreting cytokines such as IL-4, IL-10, and TGF-β, thereby delaying joint degeneration and cartilage damage; in contrast, Th1 and Th17 cell subsets mediate pro-inflammatory responses and exacerbate joint inflammation.

#### 4.3.2 Macrophages

Macrophages are heterogeneous and can be classified into M1 and M2 subtypes based on surface marker molecules (137). Among them, M1 macrophages can be activated by pro-inflammatory stimuli—such as LPS and IFN-γ—to produce pro-inflammatory cytokines, including iNOS, IL-6, IL-1β, and TNF-α. M2 macrophages can be activated by IL-4 and IL-13 and produce anti-inflammatory cytokines, such as TGF-β and IL-10. In the knee joint synovium of patients with OA, macrophages are the most abundant immune cell population (138). Because OA increases intestinal permeability, LPS binds specifically to TLR4 on macrophage surfaces, promoting polarization toward M1 macrophages and thereby activating NF-κB and other inflammatory signaling pathways—ultimately exacerbating local and systemic joint inflammation (55). It has been reported that obesity is associated with an increase in pro-inflammatory gut microbiota, accompanied by enhanced intestinal macrophage signatures and elevated systemic pro-inflammatory cytokine levels, thereby driving macrophage migration to the synovium and exacerbating joint inflammation (76). The predominant immune cells in the knee joints of OA patients are macrophages and neutrophils, which act synergistically to promote disease onset and progression (139). Importantly, the number of activated macrophages is significantly correlated with knee pain symptoms and the radiographic severity of KOA (140). Similarly, another study detected abundant activated macrophages in the synovium and joint capsule of KOA patients (141). Interestingly, metabolites produced by *Streptococcus* species can cross the intestinal barrier, activate synovial macrophages, and induce low-grade systemic inflammation, thereby exacerbating joint inflammation and tissue damage (10). To sum up, macrophages serve as a key immune hub connecting intestinal flora dysfunction and the progression of osteoarthritis. Further study of their specific molecular mechanisms in the gut microbiota and joints is expected to provide a theoretical basis for the targeted regulation of macrophages as therapeutic targets for osteoarthritis.

In short, OA risk factors, including aging, gender, dietary, and obesity, initially trigger systemic low-grade inflammation and intestinal barrier dysfunction, thereby inducing gut microbiota dysbiosis. This dysbiosis, in turn, exacerbates systemic inflammation via LPS translocation, reduced SCFAs levels, an elevated Th17/Treg ratio, and recruitment of pro-inflammatory macrophages, delivering a “second hit” to the joints. However, the causal relationships among these sequential events remain incompletely defined. Therefore, OA management requires not only correction of gut microbiota dysbiosis but also concurrent intervention against upstream risk factors.

## 5 Therapeutic strategies for OA targeting the gut microbiota

In recent years, with the deepening of research, the role of the gut–joint axis in the onset and progression of OA has gradually become clearer. On the one hand, the intestinal microbiota participates in human metabolic processes, and its metabolites can modulate immune system function; abnormal immune activation plays a key role in OA pathogenesis. On the other hand, impaired intestinal barrier function may allow harmful substances to enter systemic circulation, thereby contributing to joint inflammation and tissue damage. Given the unique and critical role of the gut–joint axis in OA, targeting the intestinal microbiota represents a highly promising therapeutic strategy (**Table 1**). Modulating the composition and function of the gut microbiota holds potential to ameliorate OA pathology at its root and open novel avenues for OA treatment.

**Table 1:** Therapeutic strategies targeting the gut–joint axis to modulate the gut microbiota in OA.

Interventions	Subject	Particular mechanism of action	Ref.
*Lactobacillus casei Shirota* and *Streptococcus thermophil us*	KOA patients	Alleviated OA-related symptoms by restoring intestinal microbial homeostasis and reduced hs-CRP concentrations	(142, 143)
*Saccharomyces boulardii*	KOA patients	Improved pain symptoms and reduced serum levels of hs-CRP and malondialdehyde	(144)
*Streptococcus thermophilus, Lactobacillus pentosus, and* GABA	ACLT-induced OA mouse model	Increased GABA concentration, inhibited pro-inflammatory cytokine production, protected cartilage, and suppress OA progression	(145)
*Lactobacillus acidophilus*	Experimental murine OA model	Ameliorated OA-associated gut microbiota dysbiosis, alleviated joint pain, and protected cartilage structure	(146)
*Lactobacillus kefiranofaciens*	MIA-induced OA model	Increased the abundance of beneficial bacteria, elevated SCFAs production, enhanced intestinal barrier function, alleviated oxidative stress, and thereby reduced joint inflammation.	(119)
Propionate	MIA-induced OA rat model	Inhibited the progression of by restoring intestinal barrier function, modulated gut microbiota composition, alleviated pain, protected cartilage structure, and downregulated inflammatory markers	(113)
Butyrate	KOA patients	Enhanced intestinal barrier integrity and reduced circulating levels of LBP	(150)
Miya	ACLT-induced OA rat model	Ameliorated OA-associated gut dysbiosis and increased the relative abundances of *Lactobacillus*, *Clostridium*, *Coprococcus*, and *Oscillospira*	(101)
*Lycium barbarum* polysaccharide	DMM-induced OA model	Suppressed ferroptosis in chondrocytes and increased the *Lactobacillus*/*Alistipes* ratio	(151)
Mulberry aqueous extract	DMM-induced KOA rat model	Increased the abundance of *Lactobacillus johnsonii* in the gut, inhibited M1 macrophage polarization, and promoted M2 macrophage polarization	(121)
Liubao tea	DMM-induced OA mouse model	Restored intestinal microbiota homeostasis by increasing microbial alpha diversity, improving cartilage structure, and suppressing systemic inflammatory responses	(153)
Pterostilbene	Octacalcium phosphate-induced OA mouse model	Regulated the structure of the gut microbiota and inhibited NLRP3 inflammasome activation by increasing the abundance of beneficial bacteria while decreasing inflammation-associated bacteria	(154)
Isopsoralen	DMM-induced OA mouse model	Inhibited the activation of the MAPK and NF-κB signaling pathways by increasing the abundance of beneficial bacteria and SCFAs	(120)
Sclareol	KOA rat model	Reduced the relative abundances of the *Prevotellaceae ga6a1* and *Corynebacterium* and suppressed necroptosis in synovial macrophages	(155)
Green-lipped mussel, glucosamine	KOA patients	Reduced the relative abundances of *Clostridium* and *Staphylococcus*, whereas increased the relative abundances of *Lactobacillus*, *Streptococcus*, and *Eubacterium*	(159)
Gold nanoparticles	ACLT-induced murine OA model	Increased the relative abundances of *Akkermansia* and *Lactobacillus*, enhanced butyrate production	(162)
*Bifidobacterium animalis* subsp. *lactis* Probio-M8 and chondroitin sulfate	KOA patients	Increased the relative abundances of beneficial bacteria, including *Agathobaculum butyriciproducens*, *Bacteroides stercoris*, *Bifidobacterium animalis*, *Roseburia hominis*, and *Ruminococcus bromii*	(163)
Platelet-rich plasma	MIA-induced OA rat model	Restored intestinal barrier function, increased the abundance of *Ligilobacillus murinus*, and reduced joint inflammation.	(166)
Moxibustion	ACLT-induced murine OA model	Reduced the relative abundance of *Ruminococcus* and *Proteobacteria*	(100)
Electroacupuncture	KOA patients	Modulated the composition of the gut microbiota and increased the relative abundance of *Agathobacter* and *Lachnoclostridium*	(170)
Electroacupuncture combined with massage	KOA patients	Modulated of the gut microbiota composition, improved intestinal barrier integrity, and suppressed systemic inflammatory responses	(171)

ACLT, anterior cruciate ligament transection; DMM, medial meniscus destabilization; GABA, gamma-aminobutyric acid; hs-CRP, high-sensitivity C-reactive protein; KOA, knee osteoarthritis; LBP, lipopolysaccharide-binding protein; MLI, meniscal and ligamentous injury; MIA, monoiodoacetate; MAPK, mitogen-activated protein kinase; NLRP3, NOD-like receptor protein 3; OA, Osteoarthritis; SCFAs: short-chain fatty acids; NF–κB, nuclear factor kappa-light-chain-enhancer of activated B cells.

### 5.1 Probiotics therapies

A growing body of randomized, double-blind, placebo-controlled clinical trials has demonstrated that oral probiotic supplementation (*Lactobacillus casei Shirota* and *Streptococcus thermophilus*) alleviates OA-related symptoms by restoring intestinal microbial homeostasis and reducing high-sensitivity C-reactive protein (hs-CRP) concentrations (142, 143). In another randomized, triple-blind, placebo-controlled trial, the probiotic *Saccharomyces boulardii* improved pain symptoms in patients with KOA and reduced serum levels of hs-CRP and malondialdehyde (144). In the ACLT-induced OA mouse model, *Streptococcus thermophilus*, *Lactobacillus pentosus*, and gamma-aminobutyric acid (GABA) all increased GABA concentration, inhibited pro-inflammatory cytokine production, protected cartilage, and suppressed OA progression (145). Moreover, *Lactobacillus acidophilus* significantly ameliorated OA-associated gut microbiota dysbiosis, alleviated joint pain, and protected cartilage structure (146). Similarly, *Latilactobacillus sakei* LB-P12 can also reduce joint inflammation and inhibit OA progression (147). In a MIA-induced OA model, *Lactobacillus kefiranofaciens* increased the abundance of beneficial bacteria, including *Ruminococcaceae* and *Lachnospiraceae*, elevated SCFAs production, enhanced intestinal barrier function, alleviated oxidative stress, and thereby reduced joint inflammation (119). Propionate is a major gut-related SCFAs that plays a protective role in a variety of chronic diseases (148, 149). A recent study reported that propionate inhibits the progression of MIA-induced OA model by restoring intestinal barrier function, modulating gut microbiota composition, alleviating pain, protecting cartilage structure, and downregulating inflammatory markers (113). Butyrate (another SCFAs) also improved KOA-related symptoms, primarily by enhancing intestinal barrier integrity and reducing circulating levels of LBP (150). Based on the above evidence, the therapeutic potential of probiotics and their metabolite, SCFAs, in OA is demonstrated.

### 5.2 Prebiotics therapies

Miya is a probiotic preparation derived from *Clostridium butyricum*, reported to ameliorate OA-associated gut dysbiosis and increase the relative abundances of *Lactobacillus*, *Clostridium*, *Coprococcus*, and *Oscillospira*, thereby alleviating OA-related clinical symptoms (101). In the mouse medial DMM-induced KOA model, *Lycium barbarum* polysaccharide inhibits OA progression by suppressing ferroptosis in chondrocytes and increasing the *Lactobacillus*/*Alistipes* ratio (151). Besides, Polyporus Umbellatus polysaccharide reduced the abundance of *Odoribacter* and *Alistipes*, but increased the abundance of *Cryptobacteria* and *Lactobacillus*, and inhibited Wnt/β-catenin and NF-κB signaling pathways to relieve joint inflammation (152). Mulberry aqueous extract also ameliorated synovial inflammation and cartilage lesions in the DMM-induced KOA rat model, primarily through increasing the abundance of *Lactobacillus johnsonii* in the gut, inhibiting M1 macrophage polarization, and promoting M2 macrophage polarization (121). Liubao tea also inhibited the progression of DMM-induced OA, primarily by restoring intestinal microbiota homeostasis—particularly by increasing microbial alpha diversity, improving cartilage structure, and suppressing systemic inflammatory responses (153). As a natural product, pterostilbene exhibits anti-inflammatory and anti-obesity effects. It has been reported that pterostilbene regulates the structure of the gut microbiota and inhibits NLRP3 inflammasome activation by increasing the abundance of beneficial bacteria while decreasing inflammation-associated bacteria, thereby delaying OA progression (154). Additionally, another natural product, isopsoralen, inhibits the activation of the mitogen-activated protein kinase (MAPK) and NF-κB signaling pathways by increasing the abundance of beneficial bacteria, including *Blautia* and *Allobaculum*, and their metabolites, SCFAs, thereby reducing joint inflammatory responses and alleviating OA (120). Sclareol exhibits potent anti-inflammatory, antibacterial, and antioxidant activities. In the KOA model, Sclareol reduced the relative abundances of the *Prevotellaceae ga6a1 group* and *Corynebacterium* and suppressed necroptosis in synovial macrophages, thereby alleviating OA-associated synovial inflammation (155). Green-lipped mussels (GLM) are a well-established natural source of long-chain omega-3 polyunsaturated fatty acids, whose bioactive metabolites suppress key pro-inflammatory mediators, thereby attenuating joint inflammation and associated pain (156–158). In a randomized, unblinded, controlled clinical trial, both GLM and glucosamine interventions significantly reshaped the gut microbiota structure in patients with OA: the relative abundances of *Clostridium* and *Staphylococcus* were significantly reduced, whereas those of *Lactobacillus*, *Streptococcus*, and *Eubacterium* were significantly increased; concurrently, both interventions were associated with significant improvements in the Gastrointestinal Symptom Rating Scale (GSRS) scores and OA-related clinical symptoms, suggesting a potential synergistic link between microbiota modulation and symptom alleviation (159). Inulin, a prebiotic supplement, increases levels of butyric acid and glucagon-like peptide-1 to improve joint pain in patients with KOA (160, 161). Additionally, gold nanoparticles have been reported to increase the relative abundances of *Akkermansia* and *Lactobacillus*, enhance butyrate production, and thereby attenuate OA progression (162). The combination of *Bifidobacterium animalis* subsp. *lactis* Probio-M8 and chondroitin sulfate increases the relative abundances of beneficial bacteria, including *Agathobaculum butyriciproducens*, *Bacteroides stercoris*, *Bifidobacterium animalis*, *Roseburia hominis*, and *Ruminococcus bromii*, while decreasing that of *Dorea formicigenerans*, thereby significantly alleviating KOA-associated symptoms (163). All of the above substances have potential as prebiotics, suggesting that prebiotics may become DMOADs.

However, intervention with colistin and *Escherichia coli* can lead to gut microbiota dysbiosis, thereby exacerbating OA progression. These interventions primarily alter the composition of the gut microbiota, resulting in downregulation of intestinal tight junction proteins, including ZO-1 and Occludin, which increases intestinal permeability, enhances LPS translocation, and promotes M1 macrophage polarization (164). In summary, oral therapies represented by probiotics, prebiotics, and natural products can reshape gut microbiota composition, increase SCFAs production, enhance intestinal barrier integrity, and reduce LPS translocation, thereby inhibiting the TLR4/NF-κB inflammatory signaling pathway, and promoting macrophage polarization toward the M2 phenotype. These effects collectively improve systemic low-grade inflammation and clinical manifestations in patients with OA, indicating broad therapeutic potential. However, current clinical evidence remains limited by small sample sizes, short intervention durations, insufficient standardization of bacterial strains and natural product components, and the absence of stratification based on baseline gut microbiota composition and immune–metabolic phenotypes, leading to substantial interstudy heterogeneity in treatment efficacy. Therefore, future studies should optimize oral therapy regimens, including strain selection, dosing, treatment duration, and combinatorial strategies, within precisely defined patient subgroups, and conduct rigorously designed, large-scale, multicenter randomized controlled trials to clarify its clinical positioning and translational value in modulating the gut–immune axis in OA.

### 5.3 Other therapies

Platelet-rich plasma (PRP) is a platelet concentrate rich in multiple growth factors (165). A recent study reported that, in a MIA-induced rat model of KOA, PRP restored intestinal barrier function, specifically increased the abundance of *Ligilobacillus murinus*, and reduced joint inflammation (166). It is worth noting that transplanting the microbiota of PRP into KOA rats significantly alleviated joint swelling, repaired cartilage damage, and suppressed local inflammatory reactions in the joints. This demonstrates the therapeutic potential of FMT in OA and has been confirmed in multiple studies (120, 153, 155). Moxibustion is a cornerstone of traditional Chinese medicine (TCM) external therapy and represents a distinctive modality within the broader field of acupuncture and moxibustion. Robust evidence from clinical and preclinical studies demonstrates that moxibustion not only significantly alleviates core OA symptoms, including pain, stiffness, and functional impairment, but also attenuates key pathological processes such as cartilage degradation and joint structural deterioration (167–169). In the ACLT-induced murine OA model, moxibustion intervention markedly restored gut microbiota composition, shifting it toward that of the sham-operated control group; notably, the relative abundances of pro-inflammatory bacterial taxa, including *Ruminococcus* and *Proteobacteria*, were significantly downregulated (100). Furthermore, electroacupuncture has also been reported to improve KOA, primarily by modulating the composition of the gut microbiota, shifting it toward a healthier profile, and particularly by increasing the relative abundance of *Agathobacter* and *Lachnoclostridium* (170). Interestingly, electroacupuncture combined with massage was superior to electroacupuncture alone in alleviating joint pain, improving joint stiffness, and enhancing joint function in KOA patients. The underlying mechanism may involve more pronounced modulation of the gut microbiota composition, restoration of intestinal barrier integrity, and effective suppression of systemic inflammatory responses (171). Exercise intervention not only alleviates pain symptoms and improves joint mobility in patients with OA but also exerts beneficial systemic effects on host health by reshaping the gut microbiota structure, modulating its metabolic activity, and altering its key metabolite profile (172). Moderate exercise significantly increased the relative abundances of *Bacteroidales_S24-7*, *Prevotellaceae*, and *Bifidobacteriaceae*; significantly decreased the relative abundances of *Lactobacillus*, *Adlercreutzia*, *Desulfovibrionaceae*, *and Peptostreptococcaceae*; reduced the F/B ratio; attenuated LPS translocation; and thereby inhibited OA progression (99, 173).

Although therapies targeting the gut microbiota have demonstrated some clinical effectiveness, existing studies generally share common limitations, such as small sample sizes, short intervention periods, lack of long-term follow-up, and insufficient safety data on joint structural endpoints. Therefore, it is urgent to conduct large-scale, multicenter, randomized, double-blind, controlled trials in the future, integrating intestinal flora metagenomics, metabolomics, and immune cell phenotyping, and to establish a dynamic monitoring system before and after intervention while extending follow-up duration to systematically evaluate long-term efficacy and safety. In conclusion, remodeling the intestinal microbiota to ameliorate intestinal microecological dysfunction represents a potentially viable treatment strategy for OA, but its clinical translation still requires high-quality, evidence-based data.

## 6 Conclusions and future research priorities

OA is a degenerative joint disease characterized by progressive loss of anatomical structures and functional impairment. Joint pain, stiffness, deformity, and limited mobility are the main clinical manifestations, which seriously impair patients’ quality of life. Furthermore, severe pain and functional disability may even lead to permanent disability, posing a serious threat to public health and imposing a substantial economic burden. Importantly, numerous OA-associated risk factors have been identified, including advanced age, obesity, unhealthy dietary patterns, and metabolic syndrome. Therefore, early and accurate identification—and subsequent mitigation—of key OA risk factors is essential for reducing the overall disease burden. The concept of the “gut–joint axis” has more clearly elucidated the bidirectional crosstalk between the gut and joints. OA risk factors initially trigger systemic low-grade inflammation and intestinal barrier dysfunction, thereby inducing gut microbiota dysbiosis. This dysbiosis subsequently exacerbates inflammation via LPS translocation, an elevated Th17/Treg cell ratio, and infiltration of pro-inflammatory macrophages, delivering a “second hit” to the joint. A growing body of evidence indicates that intestinal dysbiosis exacerbates joint damage in arthritis. Specifically, dysbiosis can promote systemic inflammation by altering gut microbiota composition (at phylum, genus, and species levels), increasing intestinal permeability, modulating gut-derived metabolites (e.g., LPS and SCFAs), and dysregulating immune cell function, ultimately driving OA pathogenesis and progression (**Figure 3**). Given the pivotal role of intestinal dysbiosis in OA, microbiota-targeted therapeutic strategies are gaining increasing attention. In particular, probiotics and prebiotics show promise in alleviating clinical symptoms and slowing OA progression, offering new hope for OA patients. Moreover, traditional Chinese medicine interventions, including electroacupuncture and massage, have demonstrated comparable therapeutic benefits. Thus, a deeper mechanistic understanding of how gut microbiota dysbiosis contributes to OA initiation and progression will provide a robust theoretical foundation for developing gut microbiota–based DMOADs.

Understanding the mechanism of intestinal flora dysfunction in OA based on the “gut–joint axis” is a hot topic in current research. However, many issues remain unresolved. At present, most research focuses on the bacterial composition of the gut microbiota in OA patients, with a lack of systematic investigation into fungi, viruses, and other microbial community members. Future studies should systematically clarify their roles in the onset and progression of OA and elucidate their specific mechanisms, potentially providing a new paradigm for OA prevention and treatment. Current research primarily relies on correlation analyses between alterations in gut microbiota and OA clinical symptoms or related biomarkers, yet causal mechanistic insights remain insufficient. Subsequent studies should integrate metagenomic functional annotations, metabolomic profiling, and host immune response data to establish a rigorous causal verification framework, using FMT, gnotobiotic animal models, and other approaches, to delineate the precise pathways and mechanisms through which specific microbes and their metabolites influence OA pathogenesis. Moreover, significant age-related differences exist in the gut microbiota structure among OA patients, likely reflecting the combined effects of aging and disease, since the gut microbiome itself undergoes dynamic changes with age. Therefore, individualized and precision-targeted therapeutic strategies must be developed based on patient age and other pathogenic factors. Currently, gut microbiota–targeted interventions, such as probiotics, prebiotics, electroacupuncture, and moderate exercise, remain largely confined to animal experiments or small-scale clinical trials and lack standardized, systematic treatment protocols. In the future, large-scale, multicenter, randomized controlled clinical trials are urgently needed to evaluate the efficacy, safety, and applicability of these interventions and other emerging microbiota-targeting strategies for OA treatment.

## References

[1] Kloppenburg M Namane M Cicuttini F . Osteoarthritis. Lancet. (2025) 405:71–85. doi: 10.1016/s0140-6736(24)02322-5 39755397

[2] Buchanan WW Kean CA Kean WF Rainsford KD . Osteoarthritis. Inflammopharmacology. (2024) 32:13–22. doi: 10.1007/s10787-023-01223-y 37195499

[3] Dell'Isola A Recenti F Giardulli B Lawford BJ Kiadaliri A . Osteoarthritis year in review 2025: Epidemiology and therapy. Osteoarthritis Cartilage. (2025) 33:1300–6. doi: 10.1016/j.joca.2025.08.015 40914550

[4] Global, Regional, and National Burden of Osteoarthritis, 1990-2020 and Projections to 2050: A Systematic Analysis for the Global Burden of Disease Study 2021 . Global, regional, and national burden of osteoarthritis, 1990-2020 and projections to 2050: A systematic analysis for the global burden of disease study 2021. Lancet Rheumatol. (2023) 5:e508–22. doi: 10.1016/s2665-9913(23)00163-7 37675071 PMC10477960

[5] Zhang Y Han Y Sun Y Hao L Gao Y Ye J . Osteoarthritis: Molecular pathogenesis and potential therapeutic options. Signal Transduct Target Ther. (2026) 11:81. doi: 10.1038/s41392-025-02556-6 41781374 PMC12960730

[6] Zhu S Qu W He C . Evaluation and management of knee osteoarthritis. J Evid Based Med. (2024) 17:675–87. doi: 10.1111/jebm.12627 38963824

[7] Beckmann NM Villamaria EE . Interventional therapies for osteoarthritis: An update. AJR Am J Roentgenol. (2022) 219:929–39. doi: 10.2214/ajr.22.27548 35731097

[8] Perruccio AV Young JJ Wilfong JM Denise Power J Canizares M Badley EM . Osteoarthritis year in review 2023: Epidemiology & therapy. Osteoarthritis Cartilage. (2024) 32:159–65. doi: 10.1016/j.joca.2023.11.012 38035975

[9] Robinson WH Lepus CM Wang Q Raghu H Mao R Lindstrom TM . Low-grade inflammation as a key mediator of the pathogenesis of osteoarthritis. Nat Rev Rheumatol. (2016) 12:580–92. doi: 10.1038/nrrheum.2016.136 27539668 PMC5500215

[10] Boer CG Radjabzadeh D Medina-Gomez C Garmaeva S Schiphof D Arp P . Intestinal microbiome composition and its relation to joint pain and inflammation. Nat Commun. (2019) 10:4881. doi: 10.1038/s41467-019-12873-4 31653850 PMC6814863

[11] Chisari E Wouthuyzen-Bakker M Friedrich AW Parvizi J . The relation between the gut microbiome and osteoarthritis: A systematic review of literature. PloS One. (2021) 16:e0261353. doi: 10.1371/journal.pone.0261353 34914764 PMC8675674

[12] Hrncir T . Gut microbiota dysbiosis: Triggers, consequences, diagnostic and therapeutic options. Microorganisms. (2022) 10:578. doi: 10.3390/microorganisms10030578 35336153 PMC8954387

[13] Winter SE Bäumler AJ . Gut dysbiosis: Ecological causes and causative effects on human disease. Proc Natl Acad Sci USA. (2023) 120:e2316579120. doi: 10.1073/pnas.2316579120 38048456 PMC10722970

[14] Chen C Zhang Y Yao X Li S Wang G Huang Y . Characterizations of the gut bacteriome, mycobiome, and virome in patients with osteoarthritis. Microbiol Spectr. (2023) 11:e0171122. doi: 10.1128/spectrum.01711-22 36515546 PMC9927108

[15] Liu S Li G Xu H Wang Q Wei Y Yang Q . Cross-talk" between gut microbiome dysbiosis and osteoarthritis progression: A systematic review. Front Immunol. (2023) 14:1150572. doi: 10.3389/fimmu.2023.1150572 37180142 PMC10167637

[16] Jhun J Cho KH Lee DH Kwon JY Woo JS Kim J . Oral administration of Lactobacillus rhamnosus ameliorates the progression of osteoarthritis by inhibiting joint pain and inflammation. Cells. (2021) 10:1057. doi: 10.3390/cells10051057 33946919 PMC8146916

[17] Niu L Chen W Yin Z Tan H Cui J Su J . Bacterial extracellular vesicles in osteoarthritis: A new bridge of the gut-joint axis. Gut Microbes. (2025) 17:2489069. doi: 10.1080/19490976.2025.2489069 40213946 PMC12716045

[18] Wei J Zhang Y Xie C Lei G Zeng C . The gut-joint axis in osteoarthritis. Nat Rev Rheumatol. (2026) 22:345–61. doi: 10.1038/s41584-026-01378-2 42098429

[19] Yang G Bi S Zhang X Zhong J Wu L . The gut-joint axis in osteoarthritis: Integrating microbiota-driven metaflammation and immunosenescence. Int Immunopharmacol. (2026) 179:116612. doi: 10.1016/j.intimp.2026.116612 41962470

[20] Sun N Zhao Y Zhang A He Y . Gut microbiota and osteoarthritis: Epidemiology, mechanistic analysis, and new horizons for pharmacological interventions. Front Cell Infect Microbiol. (2025) 15:1605860. doi: 10.3389/fcimb.2025.1605860 40740348 PMC12307352

[21] Yang Y Hao C Jiao T Yang Z Li H Zhang Y . Osteoarthritis treatment via the Glp-1-mediated gut-joint axis targets intestinal Fxr signaling. Science. (2025) 388:eadt0548. doi: 10.1126/science.adt0548 40179178

[22] Jeyaraman M Ram PR Jeyaraman N Yadav S . The gut-joint axis in osteoarthritis. Cureus. (2023) 15:e48951. doi: 10.7759/cureus.48951 38106807 PMC10725653

[23] Longo UG Lalli A Bandini B de Sire R Angeletti S Lustig S . Role of the gut microbiota in osteoarthritis, rheumatoid arthritis, and spondylarthritis: An update on the gut-joint axis. Int J Mol Sci. (2024) 25:3242. doi: 10.3390/ijms25063242 38542216 PMC10970477

[24] Gleason B Chisari E Parvizi J . Osteoarthritis can also start in the gut: The gut-joint axis. Indian J Orthop. (2022) 56:1150–5. doi: 10.1007/s43465-021-00473-8 35813544 PMC9232669

[25] Zeissig S Frost F Haller D Stallmach A Vehreschild M Schneider KM . The role of the intestinal microbiome in inflammation and cancer. Dtsch Arztebl Int. (2025) 122:567–72. doi: 10.3238/arztebl.m2025.0142 40853338 PMC12645466

[26] Tilg H Zmora N Adolph TE Elinav E . The intestinal microbiota fuelling metabolic inflammation. Nat Rev Immunol. (2020) 20:40–54. doi: 10.1038/s41577-019-0198-4 31388093

[27] Fukasawa N Tsunoda J Sunaga S Kiyohara H Nakamoto N Teratani T . The gut-organ axis: Clinical aspects and immune mechanisms. Allergol Int. (2025) 74:197–209. doi: 10.1016/j.alit.2025.01.004 39979198

[28] Zhang Y Sun C Wang Y Zhang H Fan Y Zhao H . Targeting gut-liver-kidney axis: Microbiota-derived metabolites and therapeutic implications. Cell Commun Signal. (2026) 24:107. doi: 10.1186/s12964-025-02625-x 41530748 PMC12895596

[29] Saxami G Kerezoudi EN Eliopoulos C Arapoglou D Kyriacou A . The gut-organ axis within the human body: Gut dysbiosis and the role of prebiotics. Life (Basel). (2023) 13:2023. doi: 10.3390/life13102023 37895405 PMC10608660

[30] Huang Z Chen J Li B Zeng B Chou CH Zheng X . Faecal microbiota transplantation from metabolically compromised human donors accelerates osteoarthritis in mice. Ann Rheum Dis. (2020) 79:646–56. doi: 10.1136/annrheumdis-2019-216471 32205337 PMC7384301

[31] Yang D Chen Y Guo J Xu X Yang M Xie J . The organ-joint axes in osteoarthritis: Significant pathogenesis and therapeutic targets. Aging Dis. (2024) 16:2999–3021. doi: 10.14336/ad.2024.1223 39656496 PMC12339117

[32] Han Z Wang K Ding S Zhang M . Cross-talk of inflammation and cellular senescence: A new insight into the occurrence and progression of osteoarthritis. Bone Res. (2024) 12:69. doi: 10.1038/s41413-024-00375-z 39627227 PMC11615234

[33] Knights AJ Redding SJ Maerz T . Inflammation in osteoarthritis: The latest progress and ongoing challenges. Curr Opin Rheumatol. (2023) 35:128–34. doi: 10.1097/bor.0000000000000923 36695054 PMC10821795

[34] van den Bosch MHJ Blom AB van der Kraan PM . Inflammation in osteoarthritis: Our view on its presence and involvement in disease development over the years. Osteoarthritis Cartilage. (2024) 32:355–64. doi: 10.1016/j.joca.2023.12.005 38142733

[35] Pesesse L Sanchez C Henrotin Y . Osteochondral plate angiogenesis: A new treatment target in osteoarthritis. Joint Bone Spine. (2011) 78:144–9. doi: 10.1016/j.jbspin.2010.07.001 20851653

[36] Caliogna L Berni M Torriani C Mancuso ME Di Minno MND Brancato AM . Pathogenesis of osteoarthritis, rheumatoid arthritis, and hemophilic arthropathy: The role of angiogenesis. Haemophilia. (2024) 30:1256–64. doi: 10.1111/hae.15097 39297375 PMC11659485

[37] He M Kolhoff F Mont MA Parvizi J . Is osteoarthritis a state of joint dysbiosis? Antibiotics (Basel). (2025) 14:609. doi: 10.3390/antibiotics14060609 40558199 PMC12190058

[38] Wang Y Zhao X Liu-Bryan R . Role of Tlr2 and Tlr4 in regulation of articular chondrocyte homeostasis. Osteoarthritis Cartilage. (2020) 28:669–74. doi: 10.1016/j.joca.2020.01.011 32007503 PMC7214200

[39] Klück V Boahen CK Kischkel B Dos Santos JC Matzaraki V Boer CG . A functional genomics approach reveals suggestive quantitative trait loci associated with combined Tlr4 and Bcp crystal-induced inflammation and osteoarthritis. Osteoarthritis Cartilage. (2023) 31:1022–34. doi: 10.1016/j.joca.2023.04.011 37105395

[40] Wen Z Qiu L Ye Z Tan X Xu X Lu M . The role of Th/Treg immune cells in osteoarthritis. Front Immunol. (2024) 15:1393418. doi: 10.3389/fimmu.2024.1393418 39364408 PMC11446774

[41] Thomson A Hilkens CMU . Synovial macrophages in osteoarthritis: The key to understanding pathogenesis? Front Immunol. (2021) 12:678757. doi: 10.3389/fimmu.2021.678757 34211470 PMC8239355

[42] Wu CL Harasymowicz NS Klimak MA Collins KH Guilak F . The role of macrophages in osteoarthritis and cartilage repair. Osteoarthritis Cartilage. (2020) 28:544–54. doi: 10.1016/j.joca.2019.12.007 31926267 PMC7214213

[43] Tang S Zhang C Oo WM Fu K Risberg MA Bierma-Zeinstra SM . Osteoarthritis. Nat Rev Dis Primers. (2025) 11:10. doi: 10.1038/s41572-025-00594-6 39948092

[44] Dong Y Yan Y Zhou J Zhou Q Wei H . Evidence on risk factors for knee osteoarthritis in middle-older aged: A systematic review and meta analysis. J Orthop Surg Res. (2023) 18:634. doi: 10.1186/s13018-023-04089-6 37641050 PMC10464102

[45] Jang S Lee K Ju JH . Recent updates of diagnosis, pathophysiology, and treatment on osteoarthritis of the knee. Int J Mol Sci. (2021) 22:2619. doi: 10.3390/ijms22052619 33807695 PMC7961389

[46] Duong V Abdel Shaheed C Ferreira ML Narayan SW Venkatesha V Hunter DJ . Risk factors for the development of knee osteoarthritis across the lifespan: A systematic review and meta-analysis. Osteoarthritis Cartilage. (2025) 33:1162–79. doi: 10.1016/j.joca.2025.03.003 40174718

[47] Fan Z Yan L Liu H Li X Fan K Liu Q . The prevalence of hip osteoarthritis: A systematic review and meta-analysis. Arthritis Res Ther. (2023) 25:51. doi: 10.1186/s13075-023-03033-7 36991481 PMC10053484

[48] Yu F Zhu C Wu W . Senile osteoarthritis regulated by the gut microbiota: From mechanisms to treatments. Int J Mol Sci. (2025) 26:1505. doi: 10.3390/ijms26041505 40003971 PMC11855920

[49] McCulloch K Litherland GJ Rai TS . Cellular senescence in osteoarthritis pathology. Aging Cell. (2017) 16:210–8. doi: 10.1111/acel.12562 28124466 PMC5334539

[50] Herranz N Gil J . Mechanisms and functions of cellular senescence. J Clin Invest. (2018) 128:1238–46. doi: 10.1172/jci95148 29608137 PMC5873888

[51] Ansari MY Ahmad N Haqqi TM . Oxidative stress and inflammation in osteoarthritis pathogenesis: Role of polyphenols. BioMed Pharmacother. (2020) 129:110452. doi: 10.1016/j.biopha.2020.110452 32768946 PMC8404686

[52] Villalpando-Rodriguez GE Gibson SB . Reactive oxygen species (Ros) regulates different types of cell death by acting as a rheostat. Oxid Med Cell Longev. (2021) 2021:9912436. doi: 10.1155/2021/9912436 34426760 PMC8380163

[53] Zhang T Ding S Wang R . Research progress of mitochondrial mechanism in Nlrp3 inflammasome activation and exercise regulation of Nlrp3 inflammasome. Int J Mol Sci. (2021) 22:10866. doi: 10.3390/ijms221910866 34639204 PMC8509472

[54] Li H Yang X Song Y Zhu Q Liao Z Liang Y . Prrsv infection activates Nlrp3 inflammasome through inducing cytosolic mitochondrial DNA stress. Veterinary Microbiol. (2023) 279:109673. doi: 10.1016/j.vetmic.2023.109673 36764219

[55] Huang WR Tu JX Qiao AQ Chen LJ . Gw842166x alleviates osteoarthritis by repressing Lps-mediated chondrocyte catabolism in mice. Curr Med Sci. (2022) 42:1046–54. doi: 10.1007/s11596-022-2627-z 36178576

[56] Huang Z Kraus VB . Does lipopolysaccharide-mediated inflammation have a role in Oa? Nat Rev Rheumatol. (2016) 12:123–9. doi: 10.1038/nrrheum.2015.158 26656661 PMC4930555

[57] Peshkova M Lychagin A Lipina M Di Matteo B Anzillotti G Ronzoni F . Gender-related aspects in osteoarthritis development and progression: A review. Int J Mol Sci. (2022) 23:2767. doi: 10.3390/ijms23052767 35269906 PMC8911252

[58] Laitner MH Erickson LC Ortman E . Understanding the impact of sex and gender in osteoarthritis: Assessing research gaps and unmet needs. J Womens Health (Larchmt). (2021) 30:634–41. doi: 10.1089/jwh.2020.8828 33325792

[59] Segal NA Nilges JM Oo WM . Sex differences in osteoarthritis prevalence, pain perception, physical function and therapeutics. Osteoarthritis Cartilage. (2024) 32:1045–53. doi: 10.1016/j.joca.2024.04.002 38588890

[60] Pang H Chen S Klyne DM Harrich D Ding W Yang S . Low back pain and osteoarthritis pain: A perspective of estrogen. Bone Res. (2023) 11:42. doi: 10.1038/s41413-023-00280-x 37542028 PMC10403578

[61] Roman-Blas JA Castañeda S Largo R Herrero-Beaumont G . Osteoarthritis associated with estrogen deficiency. Arthritis Res Ther. (2009) 11:241. doi: 10.1186/ar2791 19804619 PMC2787275

[62] Yang X Yan K Zhang Q Yusufu A Ran J . Meta-analysis of estrogen in osteoarthritis: Clinical status and protective effects. Altern Ther Health Med. (2023) 29:224–30. 36150013

[63] Contartese D Tschon M De Mattei M Fini M . Sex specific determinants in osteoarthritis: A systematic review of preclinical studies. Int J Mol Sci. (2020) 21:3696. doi: 10.3390/ijms21103696 32456298 PMC7279293

[64] Siddiqui R Makhlouf Z Alharbi AM Alfahemi H Khan NA . The gut microbiome and female health. Biol (Basel). (2022) 11:1683. doi: 10.3390/biology11111683 36421397 PMC9687867

[65] Rio P Caldarelli M Chiantore M Ocarino F Candelli M Gasbarrini A . Immune cells, gut microbiota, and vaccines: A gender perspective. Cells. (2024) 13:526. doi: 10.3390/cells13060526 38534370 PMC10969451

[66] Li JY Chassaing B Tyagi AM Vaccaro C Luo T Adams J . Sex steroid deficiency-associated bone loss is microbiota dependent and prevented by probiotics. J Clin Invest. (2016) 126:2049–63. doi: 10.1172/jci86062 27111232 PMC4887186

[67] Felson DT Lawrence RC Dieppe PA Hirsch R Helmick CG Jordan JM . Osteoarthritis: New insights. Part 1: The disease and its risk factors. Ann Intern Med. (2000) 133:635–46. doi: 10.7326/0003-4819-133-8-200010170-00016 11033593

[68] Nedunchezhiyan U Varughese I Sun AR Wu X Crawford R Prasadam I . Obesity, inflammation, and immune system in osteoarthritis. Front Immunol. (2022) 13:907750. doi: 10.3389/fimmu.2022.907750 35860250 PMC9289681

[69] Lementowski PW Zelicof SB . Obesity and osteoarthritis. Am J Orthop (Belle Mead NJ). (2008) 37:148–51. 18438470

[70] Messier SP Gutekunst DJ Davis C DeVita P . Weight loss reduces knee-joint loads in overweight and obese older adults with knee osteoarthritis. Arthritis Rheum. (2005) 52:2026–32. doi: 10.1002/art.21139 15986358

[71] Wang T He C . Pro-inflammatory cytokines: The link between obesity and osteoarthritis. Cytokine Growth Factor Rev. (2018) 44:38–50. doi: 10.1016/j.cytogfr.2018.10.002 30340925

[72] Courties A Gualillo O Berenbaum F Sellam J . Metabolic stress-induced joint inflammation and osteoarthritis. Osteoarthritis Cartilage. (2015) 23:1955–65. doi: 10.1016/j.joca.2015.05.016 26033164

[73] Ley RE Turnbaugh PJ Klein S Gordon JI . Microbial ecology: Human gut microbes associated with obesity. Nature. (2006) 444:1022–3. doi: 10.1038/4441022a 17183309

[74] Huang ZY Stabler T Pei FX Kraus VB . Both systemic and local lipopolysaccharide (Lps) burden are associated with knee Oa severity and inflammation. Osteoarthritis Cartilage. (2016) 24:1769–75. doi: 10.1016/j.joca.2016.05.008 27216281 PMC5026878

[75] Goh IY Ghosh U Harasymowicz NS . Advancements in understanding the role of obesity in osteoarthritis. Connect Tissue Res. (2025) 66:345–51. doi: 10.1080/03008207.2025.2533330 40673791

[76] Schott EM Farnsworth CW Grier A Lillis JA Soniwala S Dadourian GH . Targeting the gut microbiome to treat the osteoarthritis of obesity. JCI Insight. (2018) 3:e95997. doi: 10.1172/jci.insight.95997 29669931 PMC5931133

[77] Davis S Roldo M Blunn G Tozzi G Roncada T . Influence of the mechanical environment on the regeneration of osteochondral defects. Front Bioeng Biotechnol. (2021) 9:603408. doi: 10.3389/fbioe.2021.603408 33585430 PMC7873466

[78] Kasprzyk N Nandy S Grygiel-Górniak B . Diet in knee osteoarthritis-myths and facts. Nutrients. (2025) 17:1872. doi: 10.3390/nu17111872 40507141 PMC12157890

[79] Coskun Benlidayi I . Diet in osteoarthritis. Rheumatol Int. (2021) 41:1699–700. doi: 10.1007/s00296-019-04379-5 31312886

[80] Zeng J Franklin DK Das A Hirani V . The effects of dietary patterns and food groups on symptomatic osteoarthritis: A systematic review. Nutr Diet. (2023) 80:21–43. doi: 10.1111/1747-0080.12781 36278278 PMC10092134

[81] Liu Q Hebert JR Shivappa N Guo J Tao K Zeng C . Inflammatory potential of diet and risk of incident knee osteoarthritis: A prospective cohort study. Arthritis Res Ther. (2020) 22:209. doi: 10.1186/s13075-020-02302-z 32912291 PMC7488131

[82] Silvestre MP Rodrigues AM Canhão H Marques C Teixeira D Calhau C . Cross-talk between diet-associated dysbiosis and hand osteoarthritis. Nutrients. (2020) 12:3469. doi: 10.3390/nu12113469 33198220 PMC7696908

[83] Desai MS Seekatz AM Koropatkin NM Kamada N Hickey CA Wolter M . A dietary fiber-deprived gut microbiota degrades the colonic mucus barrier and enhances pathogen susceptibility. Cell. (2016) 167:1339–1353.e21. doi: 10.1016/j.cell.2016.10.043 27863247 PMC5131798

[84] Veronese N Ragusa FS Dominguez LJ Cusumano C Barbagallo M . Mediterranean diet and osteoarthritis: An update. Aging Clin Exp Res. (2024) 36:231. doi: 10.1007/s40520-024-02883-8 39625615 PMC11614952

[85] Song J Fu J . Association between dietary index for gut microbiota and osteoarthritis in the Us population: The mediating role of systemic immune-inflammation index. Front Nutr. (2025) 12:1543674. doi: 10.3389/fnut.2025.1543674 40357032 PMC12066459

[86] Gao J Yesihati M Cheng H Li T Ding R Wang W . Association of sarcopenia and its prognostic value in symptomatic knee osteoarthritis among older people in China: The first longitudinal evidence from Charls. BMC Geriatr. (2024) 24:977. doi: 10.1186/s12877-024-05556-3 39609667 PMC11603838

[87] Wang G Li Y Liu H Yu X . Gut microbiota in patients with sarcopenia: A systematic review and meta-analysis. Front Microbiol. (2025) 16:1513253. doi: 10.3389/fmicb.2025.1513253 39911254 PMC11794218

[88] Ren HC Wang YC Cheng JB Tao HC Feng H Feng ML . Revisiting the subtle relationship between metabolic syndrome and osteoarthritis. J Inflammation Res. (2026) 19:597686. doi: 10.2147/jir.S597686 41918592 PMC13033993

[89] Dabke K Hendrick G Devkota S . The gut microbiome and metabolic syndrome. J Clin Invest. (2019) 129:4050–7. doi: 10.1172/jci129194 31573550 PMC6763239

[90] Wang PX Deng XR Zhang CH Yuan HJ . Gut microbiota and metabolic syndrome. Chin Med J (Engl). (2020) 133:808–16. doi: 10.1097/cm9.0000000000000696 32106124 PMC7147654

[91] Wang X Liu Y Sun Z Li J Lu Z Huang J . Multi-omics reveal the dysregulated gut-joint axis in knee synovitis: Data from two osteoarthritis studies in China. Adv Sci (Weinh). (2026) 13:e12020. doi: 10.1002/advs.202512020 41354462 PMC12866873

[92] Liu S Li G Zhu Y Xu C Yang Q Xiong A . Analysis of gut microbiome composition, function, and phenotype in patients with osteoarthritis. Front Microbiol. (2022) 13:980591. doi: 10.3389/fmicb.2022.980591 36504782 PMC9732244

[93] Li F Wen X Xue P Xu H Wu P Xu Z . Prevotella copri-mediated caffeine metabolism involves ferroptosis of osteoblasts in osteoarthritis. Microbiol Spectr. (2025) 13:e0157524. doi: 10.1128/spectrum.01575-24 40272163 PMC12131801

[94] Ramasamy B Magne F Tripathy SK Venugopal G Mukherjee D Balamurugan R . Association of gut microbiome and vitamin D deficiency in knee osteoarthritis patients: A pilot study. Nutrients. (2021) 13:1272. doi: 10.3390/nu13041272 33924396 PMC8069973

[95] Wang X Wu Y Liu Y Chen F Chen S Zhang F . Altered gut microbiome profile in patients with knee osteoarthritis. Front Microbiol. (2023) 14:1153424. doi: 10.3389/fmicb.2023.1153424 37250055 PMC10213253

[96] Ning Y Hu M Gong Y Huang R Xu K Chen S . Comparative analysis of the gut microbiota composition between knee osteoarthritis and Kashin-Beck disease in Northwest China. Arthritis Res Ther. (2022) 24:129. doi: 10.1186/s13075-022-02819-5 35637503 PMC9150333

[97] Wang W Liu X Nan H Li H Yan L . Specific gut microbiota and serum metabolite changes in patients with osteoarthritis. Front Cell Dev Biol. (2025) 13:1543510. doi: 10.3389/fcell.2025.1543510 40027098 PMC11868077

[98] Wei J Zhang C Zhang Y Zhang W Doherty M Yang T . Association between gut microbiota and symptomatic hand osteoarthritis: Data from the Xiangya osteoarthritis study. Arthritis Rheumatol. (2021) 73:1656–62. doi: 10.1002/art.41729 33760399 PMC8457181

[99] Hao X Zhang J Shang X Sun K Zhou J Liu J . Exercise modifies the disease-relevant gut microbial shifts in post-traumatic osteoarthritis rats. Bone Joint Res. (2022) 11:214–25. doi: 10.1302/2046-3758.114.Bjr-2021-0192.R1 35382556 PMC9057523

[100] Fu L Duan H Cai Y Chen X Zou B Yuan L . Moxibustion ameliorates osteoarthritis by regulating gut microbiota via impacting Camp-related signaling pathway. BioMed Pharmacother. (2024) 170:116031. doi: 10.1016/j.biopha.2023.116031 38113621

[101] Xu T Yang D Liu K Gao Q Liu Z Li G . Miya improves osteoarthritis characteristics via the gut-muscle-joint axis according to multi-omics analyses. Front Pharmacol. (2022) 13:816891. doi: 10.3389/fphar.2022.816891 35668932 PMC9163738

[102] Yan Y Yi X Duan Y Jiang B Huang T Inglis BM . Alteration of the gut microbiota in rhesus monkey with spontaneous osteoarthritis. BMC Microbiol. (2021) 21:328. doi: 10.1186/s12866-021-02390-0 34837955 PMC8627091

[103] Rios JL Bomhof MR Reimer RA Hart DA Collins KH Herzog W . Protective effect of prebiotic and exercise intervention on knee health in a rat model of diet-induced obesity. Sci Rep. (2019) 9:3893. doi: 10.1038/s41598-019-40601-x 30846801 PMC6405910

[104] van Dijkhuizen EHP Del Chierico F Malattia C Russo A Pires Marafon D Ter Haar NM . Microbiome analytics of the gut microbiota in patients with juvenile idiopathic arthritis: A longitudinal observational cohort study. Arthritis Rheumatol. (2019) 71:1000–10. doi: 10.1002/art.40827 30592383 PMC6593809

[105] Bonato A Zenobi-Wong M Barreto G Huang Z . A systematic review of microbiome composition in osteoarthritis subjects. Osteoarthritis Cartilage. (2022) 30:786–801. doi: 10.1016/j.joca.2021.12.006 34958936

[106] Binvignat M Fellahi S Bastard JP Rousseau A Tuffet S Courties A . Serum intestinal permeability biomarkers are associated with erosive hand osteoarthritis and radiographic severity: Results from the Digicod cohort. Osteoarthritis Cartilage. (2025) 33:736–44. doi: 10.1016/j.joca.2024.09.014 40086720

[107] Murillo-Saich JD Mannochio-Russo H Sala-Climent M Argel N Quan A Hose MK . Potential role of bile acids as a microbiome-derived mechanism in synovitis of knee osteoarthritis synovitis. Osteoarthritis Cartilage. (2026) 34:794–805. doi: 10.1016/j.joca.2026.02.011 41763351 PMC13327719

[108] Won Y Yang JI Park S Chun JS . Lipopolysaccharide binding protein and Cd14, cofactors of toll-like receptors, are essential for low-grade inflammation-induced exacerbation of cartilage damage in mouse models of posttraumatic osteoarthritis. Arthritis Rheumatol. (2021) 73:1451–60. doi: 10.1002/art.41679 33559324 PMC8362181

[109] Mukhopadhya I Louis P . Gut microbiota-derived short-chain fatty acids and their role in human health and disease. Nat Rev Microbiol. (2025) 23:635–51. doi: 10.1038/s41579-025-01183-w 40360779

[110] Han J Meng X Kong H Li X Chen P Zhang XA . Links between short-chain fatty acids and osteoarthritis from pathology to clinic via gut-joint axis. Stem Cell Res Ther. (2025) 16:251. doi: 10.1186/s13287-025-04386-3 40390010 PMC12090658

[111] Ran C Xu Y Wang Q Cao H Li D Wang Y . Gut microbiota from osteoarthritic patients without obesity aggravates osteoarthritis progression in rats by enriching acetic acid. Microb Pathog. (2025) 207:107911. doi: 10.1016/j.micpath.2025.107911 40683544

[112] Wang J Hao P Sun X Ward R Tang T Chen X . New animal model of chronic gout reproduces pathological features of the disease in humans. RMD Open. (2023) 9:e003499. doi: 10.1136/rmdopen-2023-003499 37973536 PMC10660916

[113] Han S Cho KH Na HS Jhun J Moon YM Choi JW . Propionate attenuates osteoarthritis progression by regulating the gut-joint axis. Front Immunol. (2026) 17:1717556. doi: 10.3389/fimmu.2026.1717556 41890750 PMC13012969

[114] Arpaia N Campbell C Fan X Dikiy S van der Veeken J deRoos P . Metabolites produced by commensal bacteria promote peripheral regulatory T-cell generation. Nature. (2013) 504:451–5. doi: 10.1038/nature12726 24226773 PMC3869884

[115] Du HX Yue SY Niu D Liu C Zhang LG Chen J . Gut microflora modulates Th17/Treg cell differentiation in experimental autoimmune prostatitis via the short-chain fatty acid propionate. Front Immunol. (2022) 13:915218. doi: 10.3389/fimmu.2022.915218 35860242 PMC9289123

[116] Aoyama M Kotani J Usami M . Butyrate and propionate induced activated or non-activated neutrophil apoptosis via Hdac inhibitor activity but without activating Gpr-41/Gpr-43 pathways. Nutrition. (2010) 26:653–61. doi: 10.1016/j.nut.2009.07.006 20004081

[117] Kimura I Inoue D Maeda T Hara T Ichimura A Miyauchi S . Short-chain fatty acids and ketones directly regulate sympathetic nervous system via G protein-coupled receptor 41 (Gpr41). Proc Natl Acad Sci USA. (2011) 108:8030–5. doi: 10.1073/pnas.1016088108 21518883 PMC3093469

[118] Lan H Hong W Qian D Peng F Li H Liang C . Quercetin modulates the gut microbiota as well as the metabolome in a rat model of osteoarthritis. Bioengineered. (2021) 12:6240–50. doi: 10.1080/21655979.2021.1969194 34486477 PMC8806632

[119] Jia L Yuan J Chen Y Liang P Wu J Xie Y . Lactobacillus kefiranofaciens and its enhancement effect on the anti-inflammatory function of Cii/Gln in Mia-induced osteoarthritis by protecting the intestinal barrier and gut microecology. Int J Food Sci Nutr. (2025) 76:530–43. doi: 10.1080/09637486.2025.2508173 40401730

[120] Zhang A Li S Qiao J Zhong C Zhang Z Ye X . Isopsoralen alleviates osteoarthritis by modulating the Mapk/Nf-Kb signaling pathway and regulating the structure of gut microbiota. J Ethnopharmacol. (2026) 361:121257. doi: 10.1016/j.jep.2026.121257 41628870

[121] Zhao JA Zheng YZ Wang F Ye JM Guo YF Liang XD . Mulberry water extract alleviates osteoarthritis via Lactobacillus johnsonii-dependent bile acid restoration. Phytomedicine. (2026) 150:157679. doi: 10.1016/j.phymed.2025.157679 41421286

[122] Yu J Huang S Zhang J Su Z Chang Y Liu K . Bile acids insufficiency links perfluorooctane sulfonate-induced oxidative stress-mediated fatty liver with osteoarthritis. J Hazard Mater. (2026) 501:140750. doi: 10.1016/j.jhazmat.2025.140750 41380262

[123] Rushing BR McRitchie S Arbeeva L Nelson AE Azcarate-Peril MA Li YY . Fecal metabolomics reveals products of dysregulated proteolysis and altered microbial metabolism in obesity-related osteoarthritis. Osteoarthritis Cartilage. (2022) 30:81–91. doi: 10.1016/j.joca.2021.10.006 34718137 PMC8712415

[124] Li YS Luo W Zhu SA Lei GH . T cells in osteoarthritis: Alterations and beyond. Front Immunol. (2017) 8:356. doi: 10.3389/fimmu.2017.00356 28424692 PMC5371609

[125] Rosshirt N Trauth R Platzer H Tripel E Nees TA Lorenz HM . Proinflammatory T cell polarization is already present in patients with early knee osteoarthritis. Arthritis Res Ther. (2021) 23:37. doi: 10.1186/s13075-020-02410-w 33482899 PMC7821658

[126] Moradi B Rosshirt N Tripel E Kirsch J Barié A Zeifang F . Unicompartmental and bicompartmental knee osteoarthritis show different patterns of mononuclear cell infiltration and cytokine release in the affected joints. Clin Exp Immunol. (2015) 180:143–54. doi: 10.1111/cei.12486 25393692 PMC4367102

[127] Sanchez-Lopez E Coras R Torres A Lane NE Guma M . Synovial inflammation in osteoarthritis progression. Nat Rev Rheumatol. (2022) 18:258–75. doi: 10.1038/s41584-022-00749-9 35165404 PMC9050956

[128] Gómez-Aristizábal A Gandhi R Mahomed NN Marshall KW Viswanathan S . Synovial fluid monocyte/macrophage subsets and their correlation to patient-reported outcomes in osteoarthritic patients: A cohort study. Arthritis Res Ther. (2019) 21:26. doi: 10.1186/s13075-018-1798-2 30658702 PMC6339358

[129] Ishii H Tanaka H Katoh K Nakamura H Nagashima M Yoshino S . Characterization of infiltrating T cells and Th1/Th2-type cytokines in the synovium of patients with osteoarthritis. Osteoarthritis Cartilage. (2002) 10:277–81. doi: 10.1053/joca.2001.0509 11950250

[130] Dolganiuc A Stăvaru C Anghel M Georgescu E Chichoş B Olinescu A . Shift toward T lymphocytes with Th1 and Tc1 cytokine-secterion profile in the joints of patients with osteoarthritis. Roum Arch Microbiol Immunol. (1999) 58:249–58. 11845462

[131] Platzer H Nees TA Reiner T Tripel E Gantz S Hagmann S . Impact of mononuclear cell infiltration on chondrodestructive Mmp/Adamts production in osteoarthritic knee joints-an ex vivo study. J Clin Med. (2020) 9:1279. doi: 10.3390/jcm9051279 32354196 PMC7288002

[132] Platzer H Wellbrock M Pourbozorg G Mayakrishnan R Gantz S Khamees B . In knee osteoarthritis, the production of cytokines and metalloproteinases in presence of chondrocytes and Cd4(+) T cells depends on T cell subset: An *in vitro* analysis. Osteoarthr Cartil Open. (2025) 7:100642. doi: 10.1016/j.ocarto.2025.100642 40612887 PMC12226085

[133] Qi C Shan Y Wang J Ding F Zhao D Yang T . Circulating T helper 9 cells and increased serum interleukin-9 levels in patients with knee osteoarthritis. Clin Exp Pharmacol Physiol. (2016) 43:528–34. doi: 10.1111/1440-1681.12567 26926842

[134] Grieshaber-Bouyer R Kämmerer T Rosshirt N Nees TA Koniezke P Tripel E . Divergent mononuclear cell participation and cytokine release profiles define hip and knee osteoarthritis. J Clin Med. (2019) 8:1631. doi: 10.3390/jcm8101631 31590365 PMC6832735

[135] Kamel S Khalaf R Moness H Ahmed S . Serum and synovial fluid levels of interleukin-17a in primary knee osteoarthritis patients: Correlations with functional status, pain, and disease severity. Arch Rheumatol. (2022) 37:187–94. doi: 10.46497/ArchRheumatol.2022.7931 36017202 PMC9377169

[136] Okuyan HM Terzi MY Ozcan O Kalaci A . Association of Ucma levels in serum and synovial fluid with severity of knee osteoarthritis. Int J Rheum Dis. (2019) 22:1884–90. doi: 10.1111/1756-185x.13682 31424176

[137] Ji Y Li X Yao X Sun J Yi J Shen Y . Macrophage polarization: Molecular mechanisms, disease implications, and targeted therapeutic strategies. Front Immunol. (2025) 16:1732718. doi: 10.3389/fimmu.2025.1732718 41459510 PMC12740920

[138] Pessler F Chen LX Dai L Gomez-Vaquero C Diaz-Torne C Paessler ME . A histomorphometric analysis of synovial biopsies from individuals with Gulf War veterans' illness and joint pain compared to normal and osteoarthritis synovium. Clin Rheumatol. (2008) 27:1127–34. doi: 10.1007/s10067-008-0878-0 18414968

[139] Hsueh MF Zhang X Wellman SS Bolognesi MP Kraus VB . Synergistic roles of macrophages and neutrophils in osteoarthritis progression. Arthritis Rheumatol. (2021) 73:89–99. doi: 10.1002/art.41486 32783329 PMC7876152

[140] Kraus VB McDaniel G Huebner JL Stabler TV Pieper CF Shipes SW . Direct *in vivo* evidence of activated macrophages in human osteoarthritis. Osteoarthritis Cartilage. (2016) 24:1613–21. doi: 10.1016/j.joca.2016.04.010 27084348 PMC4992586

[141] Daghestani HN Pieper CF Kraus VB . Soluble macrophage biomarkers indicate inflammatory phenotypes in patients with knee osteoarthritis. Arthritis Rheumatol. (2015) 67:956–65. doi: 10.1002/art.39006 25544994 PMC4441094

[142] Lyu JL Wang TM Chen YH Chang ST Wu MS Lin YH . Oral intake of Streptococcus thermophil us improves knee osteoarthritis degeneration: A randomized, double-blind, placebo-controlled clinical study. Heliyon. (2020) 6:e03757. doi: 10.1016/j.heliyon.2020.e03757 32368640 PMC7184258

[143] Lei M Guo C Wang D Zhang C Hua L . The effect of probiotic Lactobacillus casei Shirota on knee osteoarthritis: A randomised double-blind, placebo-controlled clinical trial. Benef Microbes. (2017) 8:697–703. doi: 10.3920/bm2016.0207 28726510

[144] Dolatkhah N Jafari A Eslamian F Toopchizadeh V Saleh P Hashemian M . Saccharomyces boulardii improves clinical and paraclinical indices in overweight/obese knee osteoarthritis patients: A randomized triple-blind placebo-controlled trial. Eur J Nutr. (2024) 63:2291–305. doi: 10.1007/s00394-024-03428-5 38761281

[145] Amin U Jiang R Raza SM Fan M Liang L Feng N . Gut-joint axis: Oral probiotic ameliorates osteoarthritis. J Tradit Complement Med. (2024) 14:26–39. doi: 10.1016/j.jtcme.2023.06.002 38223812 PMC10785157

[146] I OS Natarajan Anbazhagan A Singh G Ma K Green SJ Singhal M . Lactobacillus acidophilus mitigates osteoarthritis-associated pain, cartilage disintegration and gut microbiota dysbiosis in an experimental murine Oa model. Biomedicines. (2022) 10:1298. doi: 10.3390/biomedicines10061298 35740320 PMC9220766

[147] Song M Kim WJ Shim J Song K . Latilactobacillus sakei Lb-P12 ameliorates osteoarthritis by reducing cartilage degradation and inflammation via regulation of Nf-Kb/Hif-2α pathway. J Microbiol Biotechnol. (2025) 35:e2504013. doi: 10.4014/jmb.2504.04013 40329628 PMC12089955

[148] Corte-Iglesias V Saiz ML Andrade-Lopez AC Salazar N Bernet CR Martin-Martin C . Propionate and butyrate counteract renal damage and progression to chronic kidney disease. Nephrol Dial Transplant. (2024) 40:133–50. doi: 10.1093/ndt/gfae118 38794880 PMC11852269

[149] Xu Y Qiu Z Gu C Yu S Wang S Li C . Propionate alleviates itch in murine models of atopic dermatitis by modulating sensory Trp channels of dorsal root ganglion. Allergy. (2024) 79:1271–90. doi: 10.1111/all.15998 38164798

[150] Karim A Khan HA Ahmad F Qaisar R . Butyrate (short-chain fatty acid) alleviates lipopolysaccharide-binding proteins and improves physical function in knee osteoarthritis patients. Int J Biol Macromol. (2025) 307:142017. doi: 10.1016/j.ijbiomac.2025.142017 40081693

[151] Meng T Wang J Hou N Wang Y Gu Y Lu M . Lycium barbarum polysaccharide alleviates osteoarthritis progression in mice Dmm model by inhibiting chondrocytes ferroptosis and promoting the gut Lactobacillus/Alistipes ratio. Mol Biol Rep. (2025) 52:1021:1021. doi: 10.1007/s11033-025-11141-7 41075115

[152] Zheng Z Dai J Pan X Xiang J You P Zhang H . Polyporus umbellatus polysaccharide ameliorates rheumatoid arthritis via inhibiting inflammation and regulating gut microbiota. Int J Biol Macromol. (2025) 318:145306. doi: 10.1016/j.ijbiomac.2025.145306 40527375

[153] Le G Wen R Huang Z Fang H Zheng J Wang Y . Integrating network pharmacology, microbiomics, and metabolomics to uncover the therapeutic effect of Liubao tea on osteoarthritis. Front Immunol. (2026) 17:1746350. doi: 10.3389/fimmu.2026.1746350 41798939 PMC12960094

[154] Lee YC Chang YT Cheng YH Pranata R Hsu HH Chen YL . Pterostilbene protects against osteoarthritis through Nlrp3 inflammasome inactivation and improves gut microbiota as evidenced by *in vivo* and *in vitro* studies. J Agric Food Chem. (2024) 72:9150–63. doi: 10.1021/acs.jafc.3c09749 38624135 PMC11046483

[155] Jie L Liu J Liu Y Zhang K Shen X Xu B . Sclareol alleviates synovial inflammation in knee osteoarthritis by regulating sphingolipid metabolism along the gut-bone axis. Phytomedicine. (2025) 149:157563. doi: 10.1016/j.phymed.2025.157563 41273871

[156] Yang S Min HK Park JS Na HS Cho ML Park SH . A green-lipped mussel prevents rheumatoid arthritis via regulation of inflammatory response and osteoclastogenesis. PloS One. (2023) 18:e0280601. doi: 10.1371/journal.pone.0280601 36662733 PMC9858385

[157] Gibson RG Gibson SL . Green-lipped mussel extract in arthritis. Lancet. (1981) 1:85. doi: 10.1016/s0140-6736(81)91815-8 6110063

[158] Kampa N Kaenkangploo D Jitpean S Srithunyarat T Seesupa S Hoisang S . Evaluation of the comparative efficacy of green lipped mussel plus krill oil extracts (Eab-277), Biota orientalis extracts or NSAIDs for the treatment of dogs with osteoarthritis associated pain: A blinded, placebo-controlled study. Front Vet Sci. (2024) 11:1464549. doi: 10.3389/fvets.2024.1464549 39450405 PMC11500327

[159] Coulson S Butt H Vecchio P Gramotnev H Vitetta L . Green-lipped mussel extract (Perna canaliculus) and glucosamine sulphate in patients with knee osteoarthritis: Therapeutic efficacy and effects on gastrointestinal microbiota profiles. Inflammopharmacology. (2013) 21:79–90. doi: 10.1007/s10787-012-0146-4 22821424

[160] Kouraki A Franks S Vijay A Kurien T Taylor MA Smith SL . Effect of prebiotic supplementation with and without physiotherapy on pain and pain sensitivity in people with knee osteoarthritis. Nutrients. (2026) 18:714. doi: 10.3390/nu18050714 41829888 PMC12986947

[161] Fortuna R Wang W Mayengbam S Tuplin EWN Sampsell K Sharkey KA . Effect of prebiotic fiber on physical function and gut microbiota in adults, mostly women, with knee osteoarthritis and obesity: A randomized controlled trial. Eur J Nutr. (2024) 63:2149–61. doi: 10.1007/s00394-024-03415-w 38713231

[162] Deng Z Yang C Xiang T Dou C Sun D Dai Q . Gold nanoparticles exhibit anti-osteoarthritic effects via modulating interaction of the "microbiota-gut-joint" axis. J Nanobiotechnol. (2024) 22:157. doi: 10.1186/s12951-024-02447-y 38589904 PMC11000357

[163] Wang K Wang H Zhao Z Shen X Zhao J Zhang H . Bifidobacterium animalis subsp. lactis Probio-M8 enhances chondroitin efficacy for knee osteoarthritis in postmenopausal women via the gut-joint axis. mSystems. (2025) 10:e0086225. doi: 10.1128/msystems.00862-25 41313018 PMC12710372

[164] Liu S Xu H Liu L Ma W Fan H Liu F . Gut microbiome dysbiosis accelerates osteoarthritis progression by inducing Ifp-Sm inflammation in "double-hit" mice. Arthritis Res Ther. (2025) 27:137. doi: 10.1186/s13075-025-03602-y 40624668 PMC12232632

[165] Glinkowski WM Gut G Śladowski D . Platelet-rich plasma for knee osteoarthritis: A comprehensive narrative review of the mechanisms, preparation protocols, and clinical evidence. J Clin Med. (2025) 14:3983. doi: 10.3390/jcm14113983 40507744 PMC12156035

[166] Yan D Li Q Wang M Nie Y Sun X Na L . Platelet-rich plasma attenuates knee osteoarthritis in rats via modulation of gut microbiota. Drug Des Devel Ther. (2026) 20:574392. doi: 10.2147/dddt.S574392 41939435 PMC13050257

[167] Chen S Liu W Liang C Liu H Wang P Fu Q . Efficacy and safety of moxibustion for knee osteoarthritis: A systematic review and meta-analysis. Complement Ther Clin Pract. (2025) 59:101979. doi: 10.1016/j.ctcp.2025.101979 40184698

[168] Chen X Fu L Huang Y Liao Q Zou B Yuan L . Moxibustion ameliorates abnormal subchondral bone remodeling by promoting Acsl1-mediated autophagy to degrade Nlrp3 in osteoarthritis. Chin Med. (2025) 20:125. doi: 10.1186/s13020-025-01182-2 40790756 PMC12337568

[169] Li Y Wu F Wei J Lao L Shen X . The effects of laser moxibustion on knee osteoarthritis pain in rats. Photobiomodul Photomed Laser Surg. (2020) 38:43–50. doi: 10.1089/photob.2019.4716 31549920 PMC6978776

[170] Wang TQ Li LR Tan CX Yang JW Shi GX Wang LQ . Effect of electroacupuncture on gut microbiota in participants with knee osteoarthritis. Front Cell Infect Microbiol. (2021) 11:597431. doi: 10.3389/fcimb.2021.597431 34671567 PMC8521167

[171] Guo X Guo L Lu QZ Xie H Chen J Su WL . Effect of electroacupuncture combined with tuina therapy on gut microbiota in patients with knee osteoarthritis. World J Gastroenterol. (2025) 31:105495. doi: 10.3748/wjg.v31.i18.105495 40496364 PMC12146932

[172] de Sire A de Sire R Petito V Masi L Cisari C Gasbarrini A . Gut-joint axis: The role of physical exercise on gut microbiota modulation in older people with osteoarthritis. Nutrients. (2020) 12:574. doi: 10.3390/nu12020574 32098380 PMC7071456

[173] Li K Liu A Zong W Dai L Liu Y Luo R . Moderate exercise ameliorates osteoarthritis by reducing lipopolysaccharides from gut microbiota in mice. Saudi J Biol Sci. (2021) 28:40–9. doi: 10.1016/j.sjbs.2020.08.027 33424281 PMC7783636

